# Study of sampling phases for body odor sampling prior to analysis by TD-GC×GC/ToFMS

**DOI:** 10.1007/s00216-025-05857-5

**Published:** 2025-04-14

**Authors:** Elsa Boudard, Lisa Fisson, Nabil Moumane, José Dugay, Jérôme Vial, Didier Thiébaut

**Affiliations:** 1 UMR CBI, Laboratoire des Sciences Analytiques, Bioanalytiques et Miniaturisation, ESPCI Paris, PSL Research University, 10 rue Vauquelin, 75231 Paris, Cedex 05, France; 2 SenseDetect Health-Care, 21 grande rue, 78240 Aigremont, France

**Keywords:** Body odor, Body volatolome, VOCs, GC×GC, Sampling

## Abstract

Body odor consists of a complex matrix of volatile organic compounds (VOCs), which has garnered increasing interest in fields like medicine for its potential in disease diagnosis. However, the field of body odor analysis is advancing slowly, partly due to a lack of standardized methodologies. Although gas chromatography–mass spectrometry (GC-MS) is widely used for VOC analysis, there is a broad range of sampling and extraction methods, leading to different or even sometimes contradictory results. To move toward standardized procedures, this study compares five sampling phases for direct body odor sampling in terms of analytical cleanliness and VOC trapping/release efficiency: gauze, glass beads, PowerSorb^®^, Getxent^®^ microtubes, and passive sampling pillows (PSP). Thermodesorption was employed to simplify the protocol and minimize contamination or sample loss, which often occurs during multistep processes. Given the matrix’s complexity and the need to detect trace-level compounds, comprehensive two-dimensional gas chromatography coupled with time-of-flight mass spectrometry (GC×GC/ToFMS) was used to achieve high sensitivity and peak capacity. PSP and PowerSorb^®^ demonstrated the best performance, with mean recovery yields of 95% and 71%, respectively, and 22% and 10% variability, ensuring good repeatability. These findings, initially obtained under simulated conditions with a synthetic mixture, were validated with real body odor samples, with an optimal sampling duration estimated between 30 min and 1 h. This study not only highlights these effective sampling solutions but also emphasizes the risks associated with using sorbent phases that lack adequate analytical cleanliness (i.e., clean blank) such as gauze.

**Graphical abstract:**

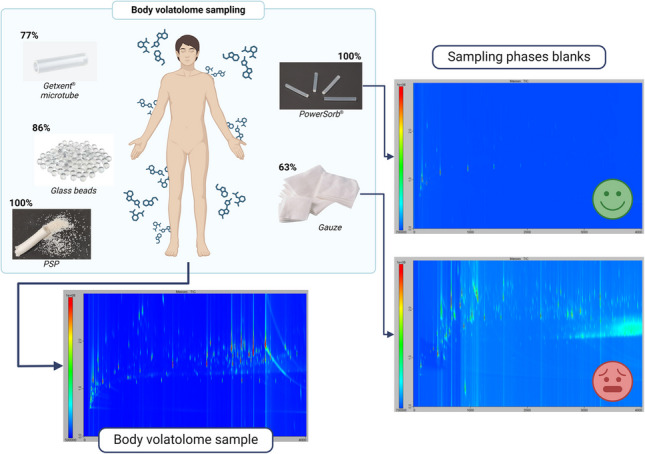

**Supplementary information:**

The online version contains supplementary material available at 10.1007/s00216-025-05857-5.

## Introduction

Body odor is a biological matrix comprising a complex mixture of over 500 volatile organic compounds (VOCs) of varying polarity and volatility. These VOCs are released from the body and are found at levels of parts per million (ppm) or billion (ppb) [1, 2]. The composition of the body volatolome can be influenced by numerous factors, including genetics, environments, diet, or metabolic state [3]. Consequently, studying the body volatolome holds significant potential for various applications. In forensics, it can serve as a biometric tool for suspect identification [4]. In medicine, it can help identify biomarkers by highlighting VOC changes related to specific pathologies, thereby facilitating noninvasive diagnosis [5]. Despite its potential, however, the application of body odor analysis in these fields is limited by the lack of standardized protocols for sampling and analysis, which hinders the comparison and reproduction of results/experiments, reducing the reliability of findings.

On the basis of several literature reviews [3, 6–10], gas chromatography coupled to mass spectrometry (GC-MS) is the most commonly used analytical method for VOCs in general. According to the World Health Organization, discrimination can be done between (i) very volatile organic compounds (VVOCs) with boiling points between < 0 °C and 50–100 °C, (ii) VOCs with boiling points between 50–100 °C and 240–260 °C, and (iii) semi-volatile organic compounds (SVOCs) with boiling points up to 380–400 °C. However, the variety of sampling methods is considerable. Two main categories of approaches have been identified: indirect sampling and direct sampling [4, 11, 12]. In indirect sampling, the analytes are transported to the trapping support via a gas flow, thus never touching the skin. In contrast, direct sampling involves a sampling phase that captures the VOCs directly from the skin. The direct sampling method has been shown to be more efficient [4, 11, 12], allowing recovery of a wider range of compounds and in greater quantities. Indeed, in this case, compounds that are less volatile and have higher boiling points (> 250 °C), such as fatty acids present at the surface of the skin, can also be trapped and studied. Therefore, this study will focus on the direct sampling approach. Nonetheless, within this direct sampling category, there are numerous strategies and a variety of sampling phases.

The most commonly used method for collecting body odor samples involves sterile gauze [6, 11, 13–19]. Typically, the gauze is rubbed on the sampled body area (hand, armpit, neck, etc.) to collect the VOCs. The chemical composition of these sterile gauzes can vary depending on the brand: Kings and Dukal brands produce pure cotton gauzes, but Johnson & Johnson produces mixed gauzes made of cellulose, viscose, and polyester. As shown by Hudson-Holness [20], the chemical composition of the gauze can impact compound recovery. Polar compounds are not easily desorbed due to hydrogen bond formation, leading to low and insufficient recoveries. Similar concerns have been raised in other studies using fabrics such as shirts, which can be made of cotton, wool, Rayon, etc. In these cases, the fabric was not rubbed against the sampled body area but instead was worn or held in place for an extended period (e.g., 30 min [21], 6 h [22], or several nights [23]. A significant parameter that differentiates these studies, making them difficult to compare, was the VOC extraction method used prior to analysis, which is not standardized. For instance, Haze et al. [23] placed the shirt used for sampling in a bag, pumped air containing the VOCs into a Tenax TA column for 18 h, and then eluted the column with diethyl ether. Smeets et al. [21] removed the gauze pads from the shirt used to sample body odor and extracted the VOCs in the headspace of a vial using sorptive extraction (HSSE) before thermodesorbing the HSSE stir bar. Heartl et al. [15] and Alves Soares et al. [18] performed solid–liquid extraction (SLE) on the gauze pads followed by solvent-assisted flavor evaporation (SAFE); in some cases, even evaporation to dryness can be found [16]. Prada et al. [11] recovered VOCs using headspace solid-phase microextraction (HS-SPME) for 21 h with a polydimethylsiloxane/divinylbenzene/carboxen (PDMS/DVB/Car) fiber.

This extensive range of protocols demonstrated several common characteristics, such as lengthy workflows and multistep processes, which collectively increased the risk of analyte loss or contamination. Moreover, the recovery yields for control samples (extraction methods performed directly on liquid-synthetic mix) were quite low: a maximum of 26.8% for Prada et al. [11] and 8.7% for Hudson et al. [20]. Additionally, the utilization of evaporation to investigate volatile compounds appeared to be an inadequate approach, and the observation of predominantly heavy compounds was expected [16].

The literature has highlighted the use of other types of sampling phases, such as glass beads [24–26], polydimethylsiloxane (PDMS) patches/wristbands [12, 27–30], and commercial phases like Sorbstar^®^ [4, 31] (former name of PowerSorb^®^ from the company ActionEurope) that have the advantage of being directly thermodesorbed. Although glass beads showed a greater affinity for less volatile compounds, PDMS and PowerSorb^®^ phases exhibited versatility in their affinity for VOCs emitted by the body volatolome. Moreover, the results obtained using these phases were considered repeatable, with variability of 6% for PowerSorb^®^ [4] and between 8.2% and 9.8% for PDMS [32]. The Getxent^®^ microtube is another commercial sorbent phase reported in the literature as being used to sample body odor. However, to our knowledge, only one study has employed it alongside an analytical method, as its primary intended use is for canine detection [33, 34]. In the study by Charles et al. [33], GC-MS analysis was performed, but the method of sample introduction to the system was not described.

The lack of standardization extends beyond VOC sampling and extraction stages, affecting the conditioning of sampling phases as well. These phases require cleaning, i.e., conditioning, prior to use, but no standardized method exists. The purpose of conditioning is to enhance the reliability of results by removing as many VOCs as possible that are initially present on the sampling phase. The efficiency of this process is assessed by performing blanks of the sampling phase. Although crucial, the mention of these steps is often neglected, likely due to their perceived triviality. However, many authors highlighted the differences and the challenges between a “sterile” and an “analytically clean” sampling phase [17]. This issue was especially true for gauze, which can be conditioned by supercritical fluid extraction (SFE) or by addition of a solvent and heating [35]. For SFE, methanol and carbon dioxide were commonly used, as demonstrated by Curran et al., who were among the few to show chromatograms of blanks before and after conditioning [17]. For processes using solvent and heating, Prada et al. [11] used 1 mL of methanol, heated for 1 h at 105 °C, while Haertl et al. [15] stirred the gauze in methanol for 10 min and let it dry at room temperature, and Alves et al. [18] stirred the fabric 10 min in dichloromethane, methanol, or hexane. This last study showed that the effectiveness of the conditioning depended on the nature of the solvent and the chemical composition of the fabric. Additionally, Zhang et al. [19] found that despite conditioning, fabric blanks can still contain over 100 VOCs, with variability reaching up to 80% depending on the gauze fragment or batch analyzed. For the other sampling phases, like PDMS, conditioning can be done either by adding a solvent and heating [30] or by applying a gas flow and heating. For Wooding et al., the process lasted one night with a hydrogen flow at 100 mL/min and heating at 250 °C [29].

Finally, in this context of body odor study, comprehensive two-dimensional gas chromatography (GC×GC) is increasingly recommended and employed [3, 4, 6, 8, 36]. Franchina et al. [37] demonstrated in their review that GC×GC enhanced sensitivity by a factor of 10 to 50 relative to conventional GC. These performance improvements suggest higher standards, especially for blanks. Therefore, the aim of our study was to leverage the advantages of thermodesorption followed by comprehensive two-dimensional gas chromatography coupled to time-of-flight mass spectrometry (TD-GC×GC/ToFMS) to compare and evaluate gauze, glass beads, PowerSorb^®^, Getxent^®^ microtubes, and the passive sampling pillow (PSP, a sampling phase developed by the US Army) as sampling phases for body odor. These phases were assessed based on their blanks and their ability to trap and release VOCs during thermodesorption, using a synthetic mix. Trends related to sampling time were studied, as well as the maximum quantity of VOCs that could be trapped in the sampling phase. Finally, results obtained under simulated conditions were validated using real samples to demonstrate their applicability to real-world scenarios. This assessment was part of the effort to identify the optimal phase for sampling body odor and to develop an appropriate sampling system. The overarching objective was to advance standardization by evaluating the majority of phases used in existing literature with the most effective analytical methodology available for studying the body volatolome.

## Materials and method

### Sampling phases

Based on the literature and our own knowledge, five sampling phases were considered for this study: gauze, glass beads, PowerSorb^®^, Getxent^®^ microtubes, and the passive sampling pillow (PSP). Pictures of the five sampling phases can be found in Figure S1. The selected gauze was a sterile gauze, 100% cotton, 7.5 × 7.5 cm (high-purity). PowerSorb^®^ is a polymer stick (2 × 20 mm [diameter × length]; ActionEurope [38]). The Getxent^®^ microtube is also a polymer stick (3.8 × 30 mm [diameter × length]; Getxent - Biodesiv LLC, Switzerland). Glass beads were provided by Pr. Štěpán Urban from the University of Chemistry and Technology of Prague (Czech Republic), and each had a diameter of 4 mm. The PSP was a home-made sampling phase inspired by the work of Gore and Associates [39] and supplied by Bruce King from the analytical research department of the US Army. The PSP consisted of a porous polytetrafluoroethylene (ePTFE) tube, 4 × 35 mm (diameter × length), thickness ~ 1 mm, packed with Tenax TA powder [poly(2,6-diphenyl-p-phenylene oxide)].

### Thermodesorption

Thermodesorption was performed using the TD100-xr system (Markes International, Bridgend, UK). Stainless steel tubes (6 × 89 mm) containing the sampling phases were desorbed at 220 °C for 20 min (except for the gauze and the Getxent microtube which were desorbed at 100 °C, see the section “Blank of the sampling phases”) with a helium (Alphagaz 1, Air Liquide) flow rate of 50 mL/min into the built-in general-purpose cold trap (C4/5-C30/32 UNITY 2) at − 10 °C containing graphitized carbon. The cold trap was finally heated for 5 min at 60–100 °C/s to 320 °C with a counter-current flow of 4.2 mL/min for desorption into the GC×GC for injection using a 3-mL/min split which led to a split ratio of 3.5. Toluene D8 (TD8) was automatically injected on the trap as internal standard (IS).

### GC×GC/ToFMS

The system employed for the two-dimensional GC×GC/ToFMS consisted of a LECO Pegasus BT4D instrument (LECO, Villepinte, France). Following the process of thermodesorption, the analytes were transferred to the GC×GC system by the carrier gas, helium, at a flow rate of 1.2 mL/min. In the primary oven, the initial chromatographic separation was performed using a nonpolar Rxi-5 ms column comprising 5% diphenyl and 95% dimethyl polysiloxane with dimensions of 30 m × 0.5 mm × 0.25 μm (Restek, Lisses, France). The modulation step was facilitated by a QuadJet™ nitrogen cryogenic modulator, employing a modulation period of 3 s, which comprised two cycles of 1.5 s each, divided into 0.90 s of hot jet and 0.6 s of cold jet. For the second-dimension separation, a medium polar DB1701 14% cyanopropyl-phenyl-methylpolysiloxane column with dimensions 50 cm × 0.18 mm × 0.18 μm (Agilent) was utilized in the secondary oven. A temperature program was applied, starting at 35 °C, holding for 2 min, and ramping up at a rate of 3 °C/min until reaching 230 °C. The secondary oven temperature was maintained at 15 °C higher than that of the primary oven for optimal performance. Subsequently, the analytes were transferred to the mass spectrometer through a transfer line (31 cm × 0.18 mm) composed of deactivated silica, operating at a temperature of 250 °C.

The mass spectrometer was operated with an electron ionization source set at 70 eV and 250 °C, utilizing a scan range of 45–300 *m/z* at a scan frequency of 200 Hz.

The data acquisition and processing utilized ChromaToF software version 5.55 developed by LECO. Initially, an automatic"peak finding"was implemented with a signal-to-noise (S/N) threshold of 1000. This methodology enabled the detection of all peaks meeting the S/N specified criteria in the chromatogram. Integrations were carried out based on the predominant *m/z* value associated with each compound. Subsequent verification procedures involved manual inspection to rectify any instances of erroneously combined peaks or to eliminate any artifacts. Identifications of the compounds were accepted if the matching score with the NIST20 library exceeded 800.

### Conditioning step

PowerSorb^®^ and Getxent^®^ were supplied as preconditioned and ready to use. However, due to the very high sensitivity of our analytical system, the effectiveness of the supplier’s conditioning process was verified by performing blank analyses before use. Similarly, glass beads and the PSP were conditioned prior to shipment, but blank analyses were conducted upon receipt to check for any contamination that may have occurred during transport. For glass beads, the supplier utilized a chromosulfuric acid mixture for conditioning, immersing the beads for 24 h, followed by successive washes with water, ethanol, and hexane, and finally drying in an oven at 270 °C for 1.5 h. The PSP conditioning procedure followed by the supplier was identical to the one detailed below in this study.

After initial blank quality assessment upon receipt of the different sampling phases, individual conditioning (except for the gauze, see 3.1) was performed to further evaluate blank quality and eliminate as many VOCs as possible, potentially introduced from external sources during transport. For the glass beads, 20 beads were placed in one tube, and for the other phases, one stick per tube was used. Tubes and their contents were conditioned using the TC- 20 tube conditioner from Markes International (Bridgend, UK), allowing simultaneous conditioning of 20 tubes. A nitrogen flow of 50 mL/min was supplied to each tube, and tubes were heated at 240 °C (except for the Getxent microtube, at 100 °C, see 3.1) for 4 h. For each blank assessment, a minimum of three replicates were performed.

### Synthetic body odor mix and spiking

Given the highly variable nature of body odor, direct sampling of real body odors to assess the performance of the sampling phases was not feasible. Therefore, a synthetic mixture was prepared to encompass a wide range of volatilities and polarities, simulating body odor to evaluate the trapping and release capacity of the sampling phases. The compounds in this mixture were selected based on their volatility, polarity, and prior identification in body odor studies, specifically following Vincent Cuzuel’s thesis work [31]. The final mixture, referred to as mix 57, consisted of 57 VOCs, each at a concentration of 15 ppm, prepared in methanol (Sigma-Aldrich). The detailed composition of the mix can be found in Table S1 and included 14 alkanes, eight alkenes, five acids and esters, 12 alcohols, eight aldehydes, four aromatics, and six ketones. The boiling points of these compounds ranged from 36 °C for pentane to 369 °C for docosane.

The objective was to spike the sampling phases with the mix 57, analyze them by TD-GC×GC/ToFMS, eventually calculate recovery yields, and evaluate the variability in the obtained areas through the relative standard deviation (RSD). Spiking was performed using a calibration solution loading rig (CSLR, Markes) with N_2_ as carrier gas at a flow rate of 50 mL/min [40]. The sampling phase was placed in an empty thermodesorption tube attached to the CSLR, and 1 μL of mix 57 was injected using a syringe. After 15 s of diffusion, the gas flow was opened and maintained for 2 min and 45 s. Each sampling phase was spiked in triplicate and analyzed using the TD-GC×GC/ToFMS method described in the “Thermodesorption” and “GC×GC/ToFMS” sections.

The same procedure was performed in triplicate on conditioned commercial thermodesorption tubes packed with Tenax TA (6 x 89 mm, 35–60 mesh, Sigma-Aldrich). The spiked Tenax TA tubes served as references in order to calculate recovery yields for the PSP and PowerSorb^®^. Tenax TA was chosen due to its widespread use in sampling VOCs across various matrices, owing to its versatility and repeatability [41]. According to the Markes reference guide [42], Tenax TA is suitable for sampling VOCs ranging from C_6_ to C_30_, including aromatics, apolar and polar compounds with boiling points above 150 °C, and semi-volatiles. Preliminary tests using liquid injection of the mix 57 indicated that this method was inadequate for comparison with thermodesorption samples. Since mix 57 was prepared in methanol and the first-dimension column was nonpolar, there was insufficient peak focusing due to poor affinity between the solvent and the stationary phase, resulting in peak broadening and lower signal intensity than in the thermodesorption samples. In addition, keeping the same analytical process was deemed relevant for the comparison, given that liquid injection and thermodesorption processes involve quite different physicochemical principles, impacting the extraction yields obtained. Therefore, in order to compare only the phases and not the injection procedures, Tenax TA was selected as the reference phase for calculating recovery yields. Recovery yields were calculated for each spiked compound using the following formula:

$$Recovery\;yield\,\left(for\;compound\;i\;spiked\;on\;sampling\;phase\;y\right)= \frac{{Mean\;area\;of\;i}_{sampling\;phase\;y}}{{Mean\;area\;of\;i}_{Tenax\;TA}}\times 100$$

### Estimation of the suitable sampling time and approximation of the maximum trapping capacity of the PowerSorb

To estimate the adequate sampling time, a conditioned PowerSorb^®^ was placed inside a clean and empty glass jar (500 mL, MDTech), to which 5 μL of the mix 57 was added. The objective was therefore to introduce a fixed quantity of VOCs into the environment at *t*_0_ and then vary the time allowed for diffusion to see how long it took to achieve maximum trapping. Each jar was washed with ethanol and dried 1 h in an oven at 80 °C. Then, the jars containing the PowerSorb^®^ and the mix were left at room temperature (~ 19 °C) for the following durations: 5, 15, 30, 45, 60, and 1440 min, with three replicates per duration. After each designated period, each PowerSorb^®^ was transferred into an empty thermodesorption tube for TD-GC×GC/ToFMS analysis, as described in the “Thermodesorption” and “GC×GC/ToFMS” sections. For each sample, the sum of the areas for the 57 compounds in the mix was calculated and normalized using the TD8 area to reduce variability in sequence analysis.

To estimate the trapping capacity of the PowerSorb^®^, one conditioned PowerSorb^®^ was placed in an empty glass jar (500 mL, MDTech). Various volumes of mix 57 were added, with three repetitions performed for each volume of 5, 10, 15, 20, 25, 50, and 70 μL corresponding to total VOC masses of 4.3, 8.6, 12.8, 17.1, 21.4, 42.8, and 59.9 μg, respectively. The jars were then left at room temperature (~ 19 °C) for 30 min. This experiment aimed to estimate the maximum trapping capacity of PowerSorb^®^, expressed in micrograms of VOCs. The experiment was repeated with two PowerSorb^®^ units to study the influence of the trapping phase quantity on the recovered VOC amounts.

### Real samples

**Fig. 1:**
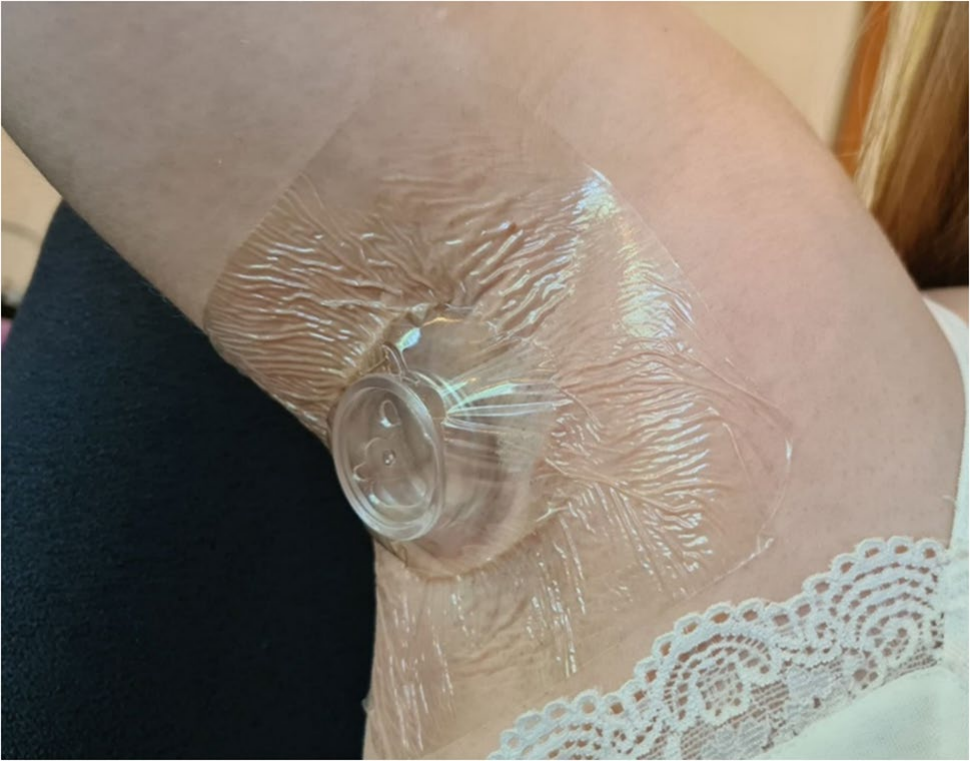
Picture of the setup used to perform axillary body odor sampling

One female (age 26) was sampled three times from both armpits using on one side PowerSorb^®^ and on the other the PSP for 1 h. Before each sampling, the armpits were cleaned using an odorless cleansing wipe (Ront Medical). For each sample, two sticks of each sampling phase were put in a small round box in polypropylene/acrylonitrile butadiene (PP/AB) and attached to the skin using a dressing film (Mepitel^®^, Molnlycke). A photo of this setup can be found in Fig. 1.

Although not optimal or particularly user-friendly, this sampling system yielded satisfactory information, and its own VOC emissions were assessed thanks to deployment of controls (results illustrated in Figure S2). These deployment controls were conducted by placing in the PP/AB box two sticks of each sampling phase (PowerSorb^®^ or PSP), and instead of fixing it to the skin, it was fastened to the inside wall of a glass jar, then left for 1 h in an oven at 40 °C.

For both the real samples and the blanks of the sampling system, at the end of the sampling hour, the sampling phases were transferred to empty thermodesorption tubes to be further analyzed by TD-GC×GC/ToFMS as described in “Thermodesorption” and “GC×GC/ToFMS” sections.

## Results and discussion

### Blank of the sampling phases

**Fig. 2:**
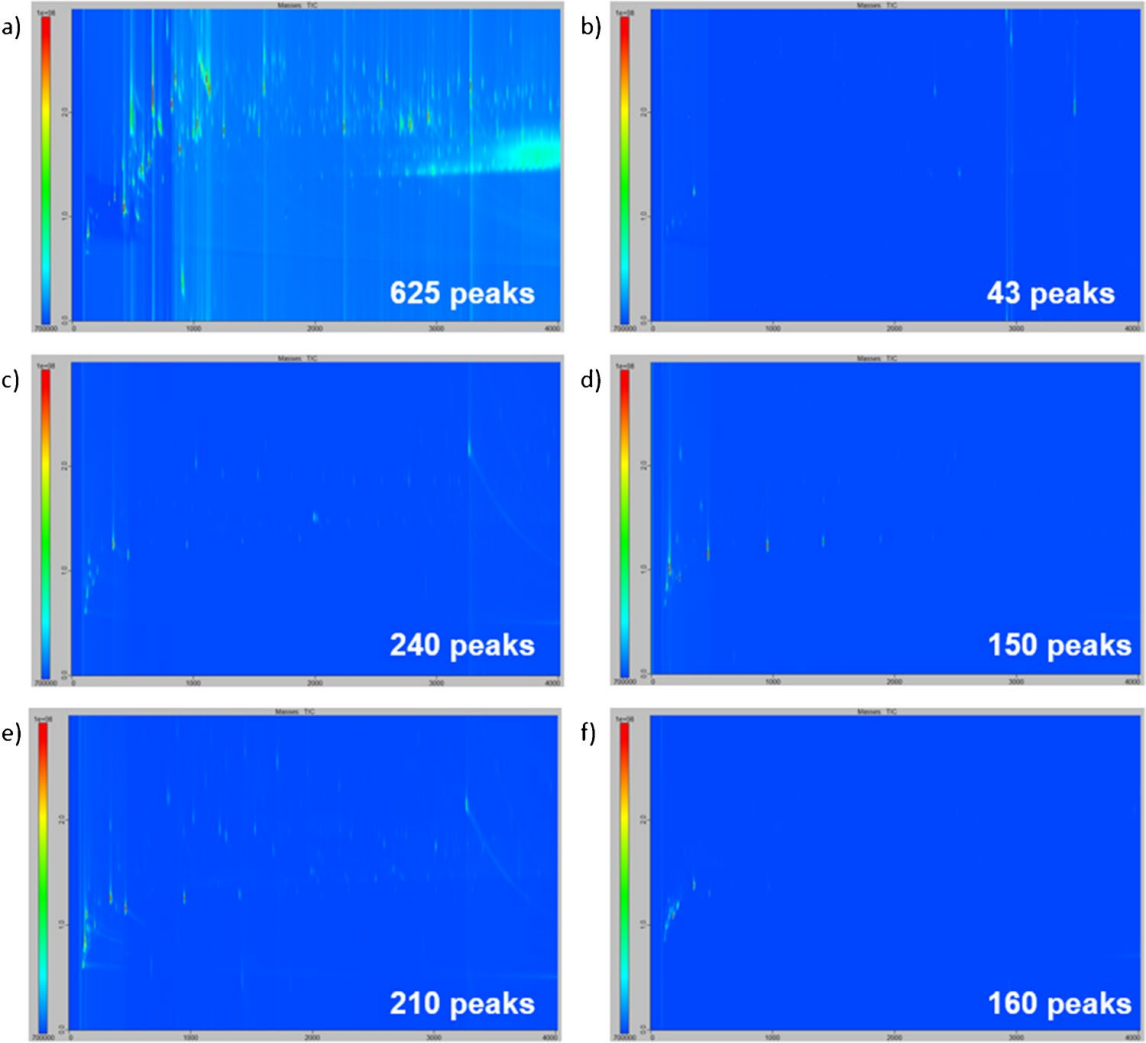
TD-GC×GC/ToFMS contour plot chromatograms of blanks for **a** raw gauze, **b** Getxent^®^ microtube conditioned for 4 h at 100 °C, **c** glass beads, **d** PowerSorb^®^, and **e** PSP conditioned for 4 h at 240 °C; **f** contour plot chromatogram of a TD-GC×GC/ToFMS system blank. All chromatograms are at the same intensity scale (from 7 × 10^6^ to 1 × 10^8^)

In the initial stage, the sampling phases were subjected to a preliminary analysis following the conditioning procedures outlined in the “Conditioning step” section. The aim of this analysis was to assess the analytical cleanliness of the various sampling phases under consideration, i.e., their VOC content before any sampling or spiking was carried out. The resulting chromatograms for the five sampling phases analyzed by TD-GC×GC/ToFMS after the implementation of a conditioning step are displayed in Fig. 2. A blank chromatogram of the analytical system depicted in Fig. 2f was obtained by analyzing an empty stainless-steel thermodesorption tube under the conditions described in the “Thermodesorption” and “GC×GC/ToFMS” sections. In Fig. 2, each spot represents a compound. Consequently, a clean sampling phase is defined as a chromatogram with a minimal number of spots. This is crucial for preventing confusion during real sampling procedures, where compounds originating from the body volatolome and compounds already present in blanks may be misidentified. As illustrated in Fig. 2, it was almost impossible to obtain a chromatogram devoid of spots, as the analytical system itself produced some VOC emissions: mainly silylated emissions linked to column bleeding or fluorinated emissions linked to the various seals in the system.

Another important point is the repeatability of the observed emissions, so that they can be monitored and, if necessary, removed when processing real samples. Firstly, the TD-GC×GC/ToFMS system itself contained an average of 160 spots with a relative standard deviation (RSD) of 52% (*n* = 7). That high variability was mostly linked to the spots observed at the beginning of the chromatogram in Fig. 1f between 0 and 500 s for the first-dimension retention time (1D-RT) and 0.5 and 1.5 s for the second-dimension retention time (2D-RT). That chromatographic area was consistently populated by numerous spots, even after the implementation of cleaning procedures on the system (blanks and bake-out procedure on the TD). Consequently, this area was rarely exploited for real samples. If needed, the area ratios can be employed to ascertain that the compound associated with the observed spot was predominant in the sample compared to the blank. For the sampling phases, the following number of peaks were detected (RSD given in parentheses, three replicates per sampling phase): 240 for glass beads (23%), 210 for PSP (2%), 150 for PowerSorb^®^ (24%), 625 for gauze (8%), and 43 for Getxent^®^ (48%). Although Getxent^®^ and gauze exhibited the lowest and highest number of peaks, respectively, it is important to note that these two phases could only be desorbed at 100 °C due to their poor ability to resist elevated temperatures. Indeed, prolonged exposure of the gauze to 100 °C resulted in browning, while the Getxent^®^ became sticky. This thermodesorption temperature, 120 °C lower compared to the other phases, had two consequences: (i) it detracted from the apparent good blank quality of the Getxent^®^ and amplified the contaminating ability of the gauze, and (ii) it limited the capacity of the Getxent^®^ to release semi-volatile compounds. Also, owing to the lack of resistance to temperature, the gauze could not be conditioned like the other phases, and Getxent^®^ was only conditioned at 100 °C. Taking this point into consideration, the best blank quality was obtained for the PowerSorb^®^, whose more intense spots (Fig. 2d, ~ 500 s, 1000 s, and 1400 s in 1D-RT) were assigned to silylated compounds, not possibly originated from body odor and therefore easy to remove from real samples.

**Fig. 3:**
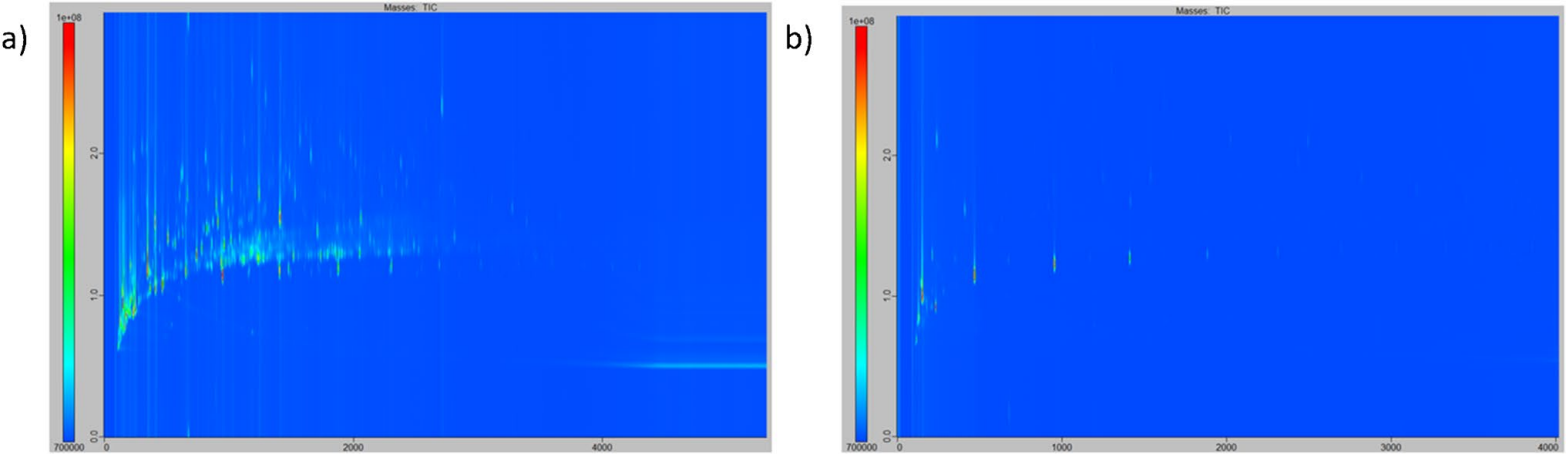
TD-GC×GC/ToFMS contour plot chromatograms of **a** PowerSorb upon receipt, and **b** PowerSorb after conditioning step (4 h at 240 °C on TC- 20, see part 2.4). Chromatograms are at the same intensity scale (from 7 × 10^6^ to 1 × 10^8^)

Secondly, Fig. 3 illustrates the impact of the conditioning step specifically for a ready-to-use phase such as PowerSorb^®^. As anticipated, the high sensitivity obtained by using TD-GC×GC/ToFMS meant a higher required efficacy level of conditioning, which could not be ensured by the manufacturer’s conditioning. The use of the method described in the “Conditioning step” section was therefore validated for conditioning phases such as the PowerSorb^®^. A total of 710 peaks were detected for analysis upon receipt for the self-described ready-to-use stick and 150 after the conditioning step. For the sum of areas, they measured from 5 × 10^10^ to 9 × 10^9^.

### Spiking

**Fig. 4:**
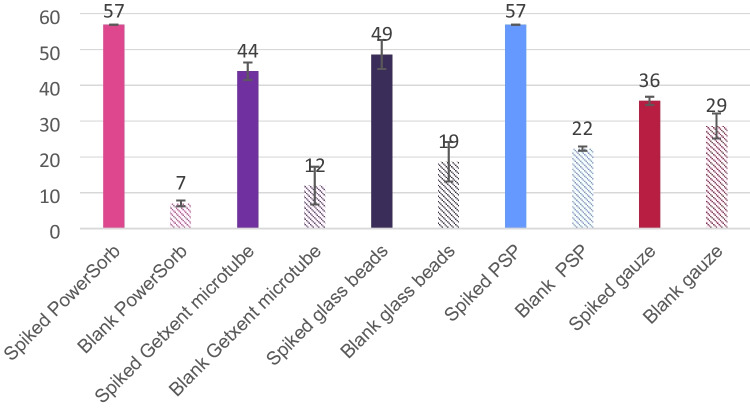
Bar chart showing the number of compounds of the mix 57 recovered after TD-GC×GC/ToFMS analysis of the five spiked sampling phases. Hatched bars show the number of compounds of the mix 57 already detected in sampling phase blanks. Error bars show the standard deviation values obtained for the three replicates

Following an assessment of the blank quality of the sampling phases, it was necessary to evaluate their capacity to trap VOCs and subsequently release them during thermodesorption. This was investigated by spiking the sampling phases using the synthetic mix 57 with the method described in the section “Synthetic body odor mix and spiking.” However, as the blanks had already been evaluated, it was deemed important to ascertain the number of the 57 compounds that were already detectable in the phase’s blanks, to have unbiased data. The obtained results are summarized in the bar chart of Fig. 4.

Figure 4 illustrates the importance of considering the blank. The most crucial and meaningful information is the differential between the spiked phase and its blank. Indeed, a sampling phase in which the blank was already highly crowded could lead to bias during spiking or sampling, as the origin of the compounds could be misinterpreted. This was the case here for the gauze which, while exhibiting the lowest number of recovered compounds (36 compounds), also contained a considerable number of the molecules considered in the mix 57 in its blanks (29 compounds). Because of its poor and biased performance, the gauze was not subject to further consideration. Conversely, it was possible to conclude that PowerSorb^®^ and PSP successfully trapped and released the 57 compounds of the mix for each replicate. This was evidenced by the fact that even the compounds detected in the blanks exhibited higher areas in the spiked samples. Nevertheless, PSP already had 22 compounds in its blanks, whereas PowerSorb^®^ had seven. For the Getxent^®^ microtube, compounds having higher boiling points like docosane, *p*-cresol, lilial, or naphthalene were not observed. This lower number of compounds (44 compounds) recovered for the Getxent^®^ microtube can be attributed to its thermodesorption temperature not exceeding 100 °C, while other phases were desorbed at 220 °C. Given this limitation, Getxent^®^ microtubes were not further considered. For the glass beads, a satisfactory number of compounds (49 compounds) were recovered. As previously highlighted in the literature [16, 25], the missing compounds were mainly volatile and apolar compounds (alpha-pinene, camphene, beta-pinene, or 3-carene).

**Fig. 5:**
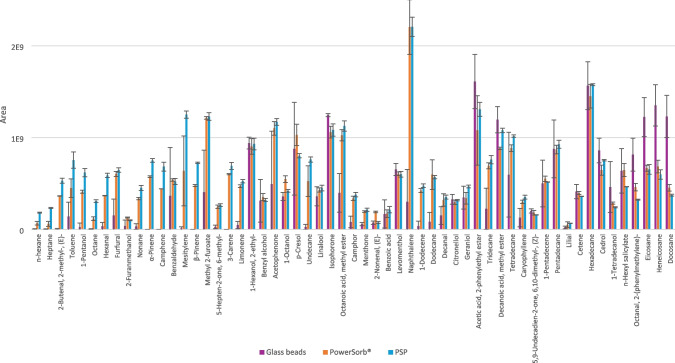
Bar chart of the average areas obtained for each compound of the mix 57 for spiked glass beads, PowerSorb^®^, and the PSP

To provide a semi-quantitative understanding of the differences between the phases’ affinities with regard to the polarity and volatility of the molecules, Fig. 5 depicts in greater detail the areas obtained for the 57 compounds of the mix, ranked in ascending order of elution in the first dimension (i.e., the more the compound is toward the right, the less volatile it is), for the PowerSorb^®^, PSP, and glass beads. This bar chart demonstrates that the areas obtained for glass beads were either negligible or null for compounds ranging from hexane to limonene. Compared to PowerSorb^®^ and PSP, the variability in peak areas was higher for glass beads across most compounds, including those trapped and released in larger quantities, such as eicosane, heneicosane, and docosane. These findings confirm the greater affinity of glass beads for less volatile and polar compounds, highlighting their limited versatility for the intended application: sampling the entire body volatolome. Additionally, Fig. 4 illustrates that the quantities recovered for PSP and PowerSorb^®^ were equivalent, with higher areas obtained with the PSP for volatile compounds (mostly hexane to beta-pinene). Regarding the variability in those areas, a mean variability of 70% was obtained for glass beads in comparison to 11% for PowerSorb^®^ and 5% for PSP. Both of these results demonstrate very satisfactory repeatability. The recovery yields were calculated for PSP and PowerSorb^®^ using the method described in the section “Synthetic body odor mix and spiking,” resulting in an average of 71% for PowerSorb^®^ and 95% for PSP. For each sampling phase, the minimum recovery yield was 28% with the PowerSorb^®^ for hexane and 68% with the PSP for lilial. The global recovery yield variability was 22% for PowerSorb and 10% for PSP. However, it is important to note that the masses of the phases were not similar (166 mg for PSP and 77 mg for PowerSorb^®^), despite the close dimensions of the sticks. Furthermore, while Tenax TA of the PSP involves adsorption phenomena, the physicochemical processes of the PowerSorb^®^ are less obvious, as the supplier did not disclose the exact nature of the polymer.

At this juncture, the outcomes were encouraging for both PowerSorb^®^ and the PSP, exhibiting promising extraction yields, good repeatability, and good versatility. However, as PSP is not commercially available, it cannot be considered a viable option for larger-scale applications at this stage. Nevertheless, its development remains a significant area of interest. Consequently, PowerSorb^®^ was selected as the optimal sampling phase for the remainder of the spiking studies.

### Estimation of the suitable sampling time and approximation of the maximum trapping capacity of the PowerSorb^®^

**Fig. 6:**
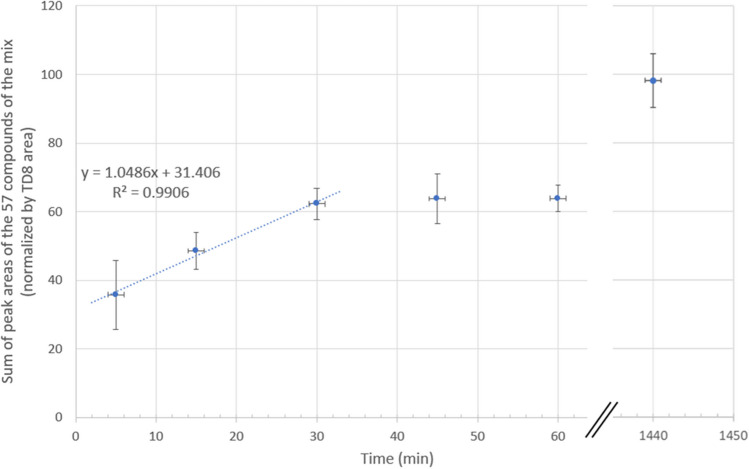
Sum of peak areas for the 57 compounds of the mix depending on the sampling time studied: 5, 15, 30, 45, 60, and 1440 min. Areas were normalized using the TD8 area. An axis break is present between the 60-min and 1440-min durations

The final objective being the sampling of VOCs coming from the body volatolome, it was interesting to assess trends regarding the sampling time and the maximum trapping capacity of the PowerSorb^®^. The assessment of the sampling time was performed in simulated conditions using the mix 57 for the PowerSorb^®^, as follows. A fixed quantity of VOCs was introduced in a closed environment at *t*_0_, and then the time allowed for diffusion was varied. After the sum of peak areas for the 57 compounds of the mix was calculated and normalized using TD8 area, these values were plotted against the sampling time, as displayed in Fig. 6. It can be seen that a plateau was reached after 30 min of exposure of the PowerSorb^®^ to the mix 57, meaning that 30 min was needed to reach equilibrium. A 24-h sampling test was also carried out and showed an increase in the total area by less than a factor of 2. Therefore, to minimize the sampling time and to obtain a qualitative and representative sample (i.e., easily observable peaks and stable values over a certain time range), a sampling time of between 30 and 60 min appeared to be reasonable. With regard to repeatability, satisfactory results were obtained for 30 and 60 min, with RSD ranging between 7% and 15%. Given that for real sampling, the total quantity of compounds was not introduced at *t*_0_ but gradually emitted by the sampled body area, a sampling time of 60 min was chosen to ensure the most representative sample possible.

**Fig. 7:**
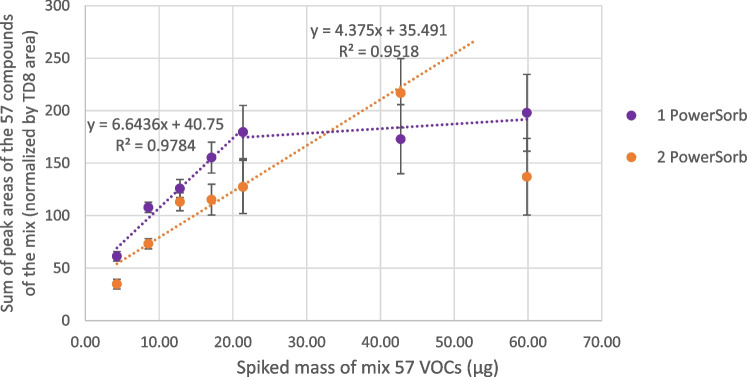
Sum of peak areas for the 57 compounds of the mix found on one and two PowerSorb^®^ units depending on the introduced mass for 30 min of exposure. Areas were normalized using the TD8 area

Then, to better understand the PowerSorb^®^ sampling phase and estimate its trapping capacity, different volumes of mix were added in a restricted environment, with a fixed diffusion time. The objective was to approximate the maximum quantity of VOCs that could be trapped by the PowerSorb^®^. Once again, the sum of peak areas for the 57 compounds of the mix was plotted, this time against the introduced mass of VOCs. As can be seen from Fig. 7, a plateau was reached when around 22 μg of VOCs had been introduced. This observed plateau meant that one PowerSorb^®^, weighing on average 77 mg, was theoretically able to trap 22 μg of VOCs. This maximum trapping capacity was evaluated with an equilibrium time of 30 min. In addition, this assessment was performed using one PowerSorb^®^; based on the study of Bicchi et al. [12], greater amounts of compounds can be obtained by increasing the available trapping surface. This was easily accomplished here by adding more PowerSorb^®^ sticks. In their study, Bicchi et al. [12] showed that the recovered quantity was tripled when the surface area of PDMS was doubled. To investigate this point, the same experiment was repeated with two PowerSorb^®^ units. As can be seen from the obtained results displayed in Fig. 7, the maximum mass of VOCs that could be trapped with two PowerSorb^®^ units increased and seemed to approach 40 μg. Even if, by looking at the directional coefficient of the linear part, it seems that equilibrium was reached more slowly, increasing the maximum quantity of VOCs trapped was a desired advantage to ensure that saturation was not reached during actual sampling. Nevertheless, while these results were intended to provide an initial understanding of how the phase works, it should be noted that further experiments would be needed to fully understand its properties related to adsorption/absorption kinetics, such as displacement effects and individual or simultaneous stick thermodesorption. Such experiments would however be beyond the scope of the present study.

### Real samples

**Fig. 8:**
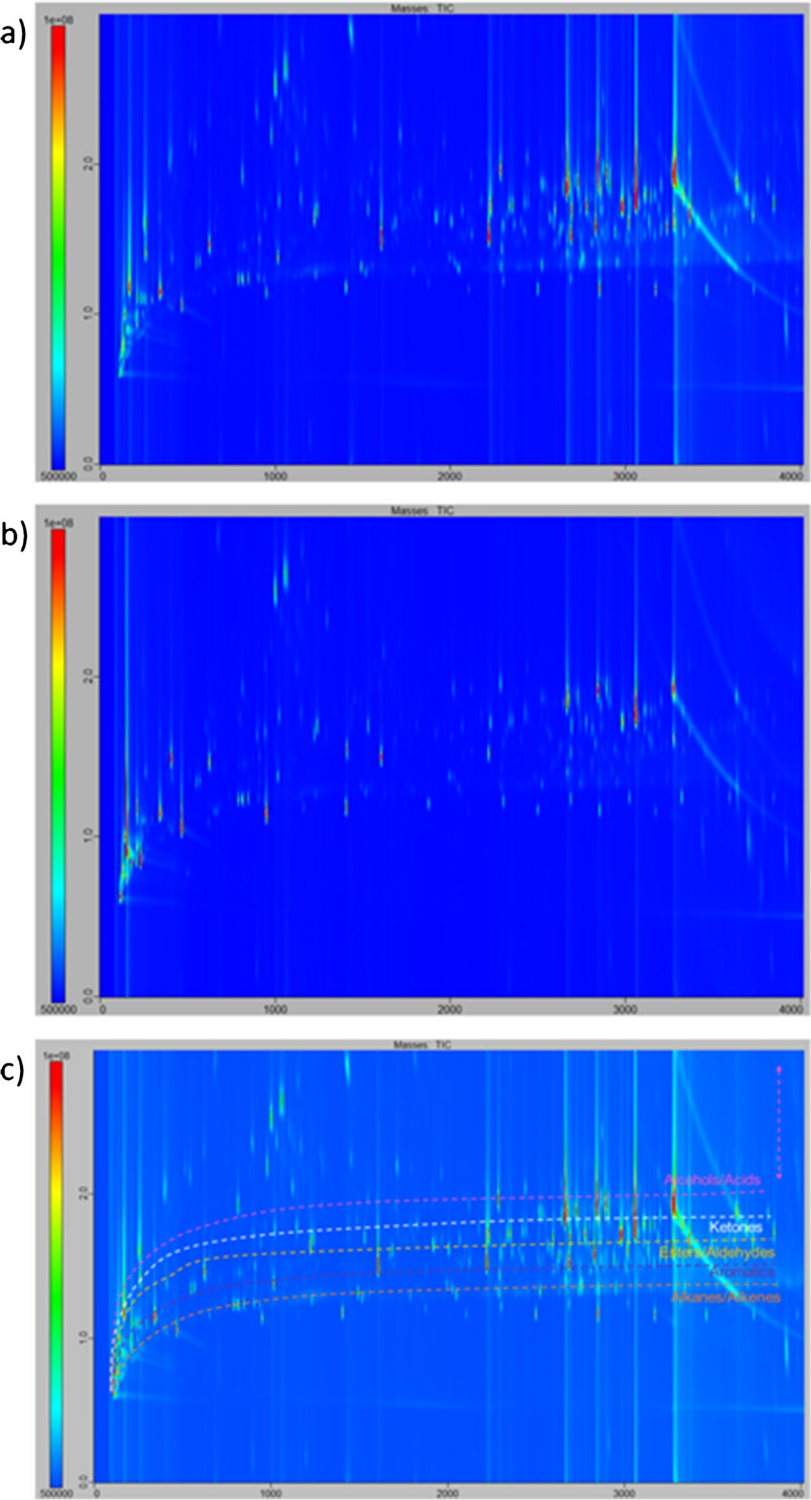
TD-GC×GC/ToFMS contour plot chromatograms of armpit body odor samples taken with the sampling phase **a** PSP and **b** PowerSorb^®^. **c** Copy of chromatogram (**a**) with a visual aid to help elucidate the results based on the stratification into chemical families along the *y* axis. Dashed lines trace the expected 2D retention time pattern of different classes of chemicals, and the vertical dashed double arrow shows where alcohol and acid compounds can be found. Chromatograms are at the same intensity scale (from 5 × 10^6^ to 1 × 10^8^)

To conclude this study on real sample applications, real sampling was conducted by sampling from the armpits of one individual for 1 h, with PSP on one side and PowerSorb^®^ on the other. Figure 8 shows the obtained chromatograms, after software subtraction of sampling device blank chromatograms (Figure S2) for armpit samples taken with PowerSorb^®^ and PSP. For the three repetitions, the mean number of detected peaks for PSP was 750 with an RSD of 17%, and for the PowerSorb^®^, 485 with an RSD of 10%. These results were consistent with the results described in the section “Spiking,” where the recovery yields for the PSP and the PowerSorb^®^ were 95% and 71%, respectively. Figure 7 also shows a quite similar pattern for the two chromatograms of body odor collected on the PSP and PowerSorb^®^, but with higher intensities for the PSP. As the separation process was conducted using a normal-phase configuration, meaning having an apolar column in the first dimension and a medium polar column in the second dimension, molecules were segregated into strata based on their polarity. This meant that when tracing along the second-dimension axis, compound families were found in the following order: hydrocarbons, aromatics, esters, ketones, aldehydes, alcohols, and acids, as shown in the visual aid in Fig. 8c. Thus, here all the chemical families previously mentioned were found, justifying the need for versatile sampling phases such as the PSP and PowerSorb^®^.

A comparison of the chromatograms obtained with the PSP and PowerSorb^®^ revealed the presence of 120 peaks in both sampling phases. However, 188 peaks were observed exclusively in the body odor chromatogram obtained with the PSP; these peaks are reported in Tables S2 and S3 with their tentative identification. These results were obtained by manually examining the presence of each peak in sampling system blanks (Figure S2). After focusing on a specific retention time window, mass spectra were compared to ensure that the same compound was considered, and then peak areas were checked. For each peak it was verified that it was not present in the blank or that its area was twice as large in the real sample. The results demonstrated that the molecules exclusively present in PSP samples were primarily compounds whose peaks were also faintly visible in the PowerSorb^®^ chromatogram. However, these peaks were not detected or validated, as their intensity was below the noise or blank emission threshold. Less than 30 peaks were observed in the body odor sample obtained with PowerSorb^®^, the majority of which were unidentified and therefore not reported here.

Ultimately, the findings substantiated the feasibility of sampling authentic body odor through the utilization of PSP or PowerSorb^®^ as the sampling phase. While greater recovery yields could be achieved with PSP, satisfactory results were also obtained with PowerSorb^®^. Although PowerSorb^®^ seemed slightly less sensitive, it had the essential characteristic of not discriminating between different chemical families. To enhance these outcomes and boost the sensitivity of PowerSorb^®^, it is essential to consider reducing the emissions from the sampling system to improve the quality of the background signal. In conclusion, the total area of the body odor sample obtained with PowerSorb^®^ was approximately 177, after normalization using the TD8 area. Based on the findings described in the previous section, it can be concluded that the two PowerSorb^®^ units captured approximately 30 μg of VOCs during a 1-h real sampling event, indicating that saturation was not reached but that two PowerSorb^®^ units were needed.

## Limitations

Our investigation on sampling phases for body odor sampling under simulated conditions has led to the identification of two phases exhibiting promising performance. However, bearing in mind the objective of moving towards standardized procedures, several limitations need to be highlighted. PowerSorb^®^ and the PSP were the two phases that demonstrated the best performance, but as the PSP is not commercially available, these results would need to be confirmed through a study based on a larger-scale manufacturing process (if available). The estimation made regarding the suitable sampling time and approximate maximum trapping capacity of the PowerSorb^®^ were intended to provide initial understanding about the sampling phase, but further assessments could be considered. For example, it is still unknown how displacement effects could affect the sampling results. Other realistic sampling conditions could be investigated, such as temporal variability of VOC release and the difference between body areas. Finally, as these sampling phases aimed to be used in a more advanced sampling device, there will be a need to confirm that their implementation in an improved sampling device does not change their performance.

## Conclusion

The aim of this study was to investigate five sampling phases considered for the collection and study of the body volatolome. The five sampling phases were sterile gauze, the Getxent^®^ microtube, glass beads, the PowerSorb^®^, and the PSP. The assessment of their analytical cleanness allowed us to confirm the enhanced need to have as low VOC emissions as possible coming from the sampling phase when using TD-GC×GC/ToFMS. The use of this sensitive and resolutive technique did not eliminate the need for a clean sampling phase. Although the greater peak capacity enables certain co-elutions to be resolved, and TD simplifies protocols by eliminating the use of solvents and removes many potential sources of contamination, it reaches its limits when the sampling phase produces many VOCs, like the gauze with more than 600 compounds emitted. Here, the gauze and the Getxent^®^ microtube had limitations regarding the thermodesorption, as they had low temperature resistance. If good results were obtained for glass beads, PowerSorb^®^, and PSP blanks, they were only made possible by the implementation of a specific preliminary conditioning step (combining heating and gas flow purging) whose effectiveness has been validated.

The ability of the sampling phases to trap and then release VOCs during thermodesorption was studied using a synthetic mix. This showed that PowerSorb^®^ and PSP were more versatile than glass beads, which had limited affinity for light and apolar compounds. Given the variety of compounds that can be found in body odor, and the need to sample everything indiscriminately for non-targeted studies, PowerSorb^®^ and Tenax are the recommended sampling phases for body odor studies. However, as the PSP is not yet commercially available yet, we decided to consider PowerSorb^®^ for initial tests. The respective recovery yields of PowerSorb^®^ and PSP were 71% and 95%, with reasonable variability of 22% and 10%. The results obtained were confirmed by real-life transposition with body odor samples collected using PowerSorb and PSP, which led to the collection of more than 450 VOCs. The estimated suitable sampling time using PowerSorb^®^ was between 30 min and 1 h. Also, the maximum trapping capacity of PowerSorb^®^ was determined to be around 22 μg for one PowerSorb^®^ unit and around 40 μg for two PowerSorb^®^ units, meaning a trapping capacity between 260 and 286 μg/g.

This study therefore appears to be a crucial step in the development of a body odor sampling system, given the central role played by the sampling phase when using TD-GC×GC/ToFMS. The next objective would be to find a way to maintain and fix this phase close to the skin without introducing additional contamination. The final objective is to provide a high-performance solution that can be deployed to conduct large-scale clinical studies aimed at identifying volatile biomarkers of pathology. In this context, achieving adequate reduction of background noise related to VOC emission produced by the sampling system itself will be the main challenge. This would allow the implementation of more advanced computing and chemometric data processing techniques, necessary for this type of study [43].

## Supplementary information

Below is the link to the electronic supplementary material.

Supplementary file1 (DOCX 1.30 MB)

## Data Availability

Data will be made available on request.
