# Supplementary material for: Study of sampling phases for body odor sampling prior to analysis by TD-GC×GC/ToFMS
Source: Anal Bioanal Chem. 2025 Apr 14;417(14):3177–90. doi: 10.1007/s00216-025-05857-5 (PMC12103381; doi:10.1007/s00216-025-05857-5)
Supplement: Supplementary file 1 — Supplementary file1 (DOCX 1.30 MB) [file 216_2025_5857_MOESM1_ESM.docx]

**Supplementary information**

Study of sampling phases for body odor sampling prior to analysis by TD‑GC×GC/ToFMS

Elsa Boudard^a,b^, Lisa Fisson^a^, Nabil Moumane^b^, José Dugay^a^, Jérôme Vial^a^, Didier Thiébaut^a^

*^a^ UMR CBI, Laboratoire des Sciences Analytiques, Bioanalytiques et Miniaturisation, ESPCI Paris, PSL Research University, 10 rue Vauquelin, 75231 Paris Cedex 05, France*

*^b^ SenseDetect Health-Care, 21 grande rue, 78240 Aigremont, France*

*Corresponding author Elsa Boudard at UMR CBI, Laboratoire des Sciences Analytiques, Bioanalytiques et Miniaturisation, ESPCI Paris, PSL Research University, 10 rue Vauquelin, 75231 Paris Cedex 05, France*

*E-mail address: elsa.boudard@espci.fr (Elsa Boudard, ORCID: 0000-0003-3844-5008)*

**Table S1** List of the 57 compounds of the mix 57

| **Compound** | **CAS number** | **Mass (g/mol)** | **^1^t_R_ (s)** | **^2^t_R_ (s)** | **Main Ions (m/z)** |
| --- | --- | --- | --- | --- | --- |
| Dodecane | 112-40-3 | 170.34 | 1511.9 | 1.273 | 57.09  71.09 |
| Docosane | 629-97-0 | 310.30 | 3692.76 | 1.370 | 57.09  71.09 |
| Eicosane | 112-95-8 | 282.50 | 3338.79 | 1.351 | 57.09  71.09 |
| Heneicosane | 629-94-7 | 296.34 | 3521.77 | 1.392 | 57.09  71.09 |
| Hexadecane | 544-76-3 | 226.44 | 2516.84 | 1.333 | 57.09  71.09 |
| Nonane | 111-84-2 | 128.26 | 650.958 | 1.182 | 57.09 |
| Octane | 111-65-9 | 114.23 | 407.974 | 1.050 | 57.09 |
| Pentadecane | 629-62-9 | 212.42 | 2288.85 | 1.373 | 57.09  71.09 |
| Tetradecane | 629-59-4 | 198.39 | 2039.87 | 1.350 | 57.09  71.09 |
| Tridecane | 629-50-5 | 184.36 | 1784.89 | 1.291 | 57.09  71.09 |
| Hexane | 110-54-3 | 86.18 | 161.99 | 0.737 | 57.09 |
| n-heptane | 142-82-5 | 100.2 | 245.984 | 0.885 | 57.09 |
| Undecane | 1120-21-4 | 156.31 | 1229.92 | 1.305 | 57.09  71.09 |
| Dodecene | 112-41-4 | 168.32 | 1487.9 | 1.347 | 55.08 |
| α-pinene | 7785-70-8 | 136.23 | 737.953 | 1.298 | 93.07  91.05 |
| β-Pinene | 19902-08-0 | 136.23 | 857.945 | 1.379 | 93.07 |
| Camphene | 79-92-5 | 136.23 | 776.95 | 1.345 | 93.07  121.09 |
| Caryophyllene | 87-44-5 | 204.35 | 2090.87 | 1.590 | 93.07  55.07  69.08 |
| D-limonene | 5989-27-5 | 136.23 | 1010.94 | 1.425 | 68.08  93.07 |
| Hexadecene | 629-73-2 | 224.43 | 2501.84 | 1.378 | 55.08  57.09 |
| Pentadecene | 13360-61-7 | 210.40 | 2267.85 | 1.369 | 55.08  57.09 |
| 1-Phenylethylester acetic acid ( 2 phenethyl acetate) | 103-45-7 | 164.20 | 1664.89 | 2.050 | 104.15 |
| 2-hydroxy, hexyl ester benzoic acid (hexyl salicylate) | 6259-76-3 | 222.30 | 2687.83 | 1.900 | 120.02 |
| Decanoic acid, methyl ester (methyl caprate) | 110-42-9 | 186.29 | 1850.88 | 1.622 | 74.05  87.04 |
| Furancarboxylic acid, methyl ester (methyl 2-furoate) | 611-13-2 | 126.11 | 860.945 | 2.044 | 95.02 |
| Octanoic acid, methyl ester (methyl octanoate) | 111-11-5 | 158.24 | 1295.92 | 1.653 | 74.05 |
| 1-octanol | 111-87-5 | 130.23 | 1139.93 | 1.919 | 55.08  69.08 |
| 1-tetradecanol | 112-72-1 | 214.39 | 2687.83 | 1.772 | 55.08  69.08  83.09 |
| 2-(2-propyl)-5-methyl-1-cyclohexanol (menthol) | 89-78-1 | 156.27 | 1430.91 | 1.903 | 81.07  55.07  67.07 |
| 2-ethyl hexanol | 104-76-7 | 130.23 | 1016.93 | 1.869 | 57.09  55.07  70.09 |
| Pentanol | 71-41-0 | 88.15 | 347.978 | 1.506 | 55.08 |
| 2-Furanmethanol (Furfuryl Alcohol) | 98-00-0 | 98.10 | 533.966 | 2.356 | 81.04  53.06  97.03 |
| 3,7-Dimethyl-1,6-Octadien-2-ol (linalol) | 78-70-6 | 154.25 | 1223.92 | 1.819 | 93.07  55.07  69.08 |
| 3,7-Dimethyl-2,6-Octadien-1-ol (geraniol) | 106-24-1 | 154.25 | 1661.89 | 1.955 | 69.08  93.06 |
| 3,7-Dimethyl-6-Octen-1-ol (citronellol) | 7540-51-4 | 156.27 | 1589.9 | 1.935 | 69.08  55.07  67.07 |
| Benzyl Alcohol | 100-51-6 | 108.14 | 1025.93 | 2.658 | 79.06  77.04  107.04 |
| Cedrol | 77-53-2 | 222.37 | 2519.84 | 1.930 | 119.08  93.06 |
| p-cresol | 106-44-5 | 108.14 | 1157.93 | 0.398 | 107.05  108.05 |
| 2-Furancarboxaldehyde (Furfural) | 98-01-1 | 96.09 | 476.969 | 1.999 | 96.02  95.01 |
| 2-Methyl-2-butenal | 497-03-0 | 84.12 | 305.98 | 1.331 | 55.08  84.06 |
| 2-nonenal | 18829-56-6 | 140.22 | 1394.91 | 2.013 | 55.07  57.05  70.05 |
| Benzaldehyde | 100-52-7 | 106.12 | 806.948 | 2.112 | 77.05  51.04  105.03 |
| Decanal | 112-31-2 | 156.27 | 1526.9 | 1.781 | 57.08  55.07 |
| Hexanal | 66-25-1 | 100.16 | 407.974 | 1.429 | 56.08  57.06 |
| α-Hexyl cinnamaldehyde | 101-86-0 | 216.32 | 2687.83 | 1.900 | 120.02 |
| α-Methyl-β-(p-tert-butylphenyl)propanal (Lilial) | 80-54-6 | 204.31 | 2348.85 | 2.046 | 189.12  131.08  147.11 |
| Benzene, 1,3,5-trimethyl (mesitylene) | 108-67-8 | 120.19 | 836.946 | 1.545 | 105.16  120.09 |
| Toluene | 108-88-3 | 92.14 | 344.978 | 1.208 | 91.07  92.06 |
| Naphthalene | 91-20-3 | 128.17 | 1451.91 | 1.964 | 128.05 |
| Carene | 13466-78-9 | 136.23 | 956.939 | 1.375 | 93.07 |
| 6-methyl-5-Hepten-2-one (sulcatone) | 110-93-0 | 126.20 | 893.943 | 1.829 | 55.08  67.07  108.09 |
| 1-Phenyl-ethanone (acetophenone) | 98-86-2 | 120.15 | 1115.93 | 2.217 | 105.03  77.04 |
| 3,5,5-trimethylcyclohex-2-en-1-one (isophorone) | 78-59-1 | 138.21 | 1274.92 | 2.326 | 82.05 |
| 6,10-Dimethyl-5,9-Undecadien-2-one | 3796-70-1 | 194.32 | 2126.86 | 1.872 | 69.08 |
| Menthone | 14073-97-3 | 154.25 | 1373.91 | 1.943 | 69.07  55.07  112.08 |
| Camphre | 76-22-2 | 152.23 | 1343.91 | 2.048 | 95.08  55.07  81.07 |

**Fig. S1** Pictures of the 5 studied sampling phases, from left to right: gauze, glass beads, PowerSorb®, Getxent® microtube, PSP.


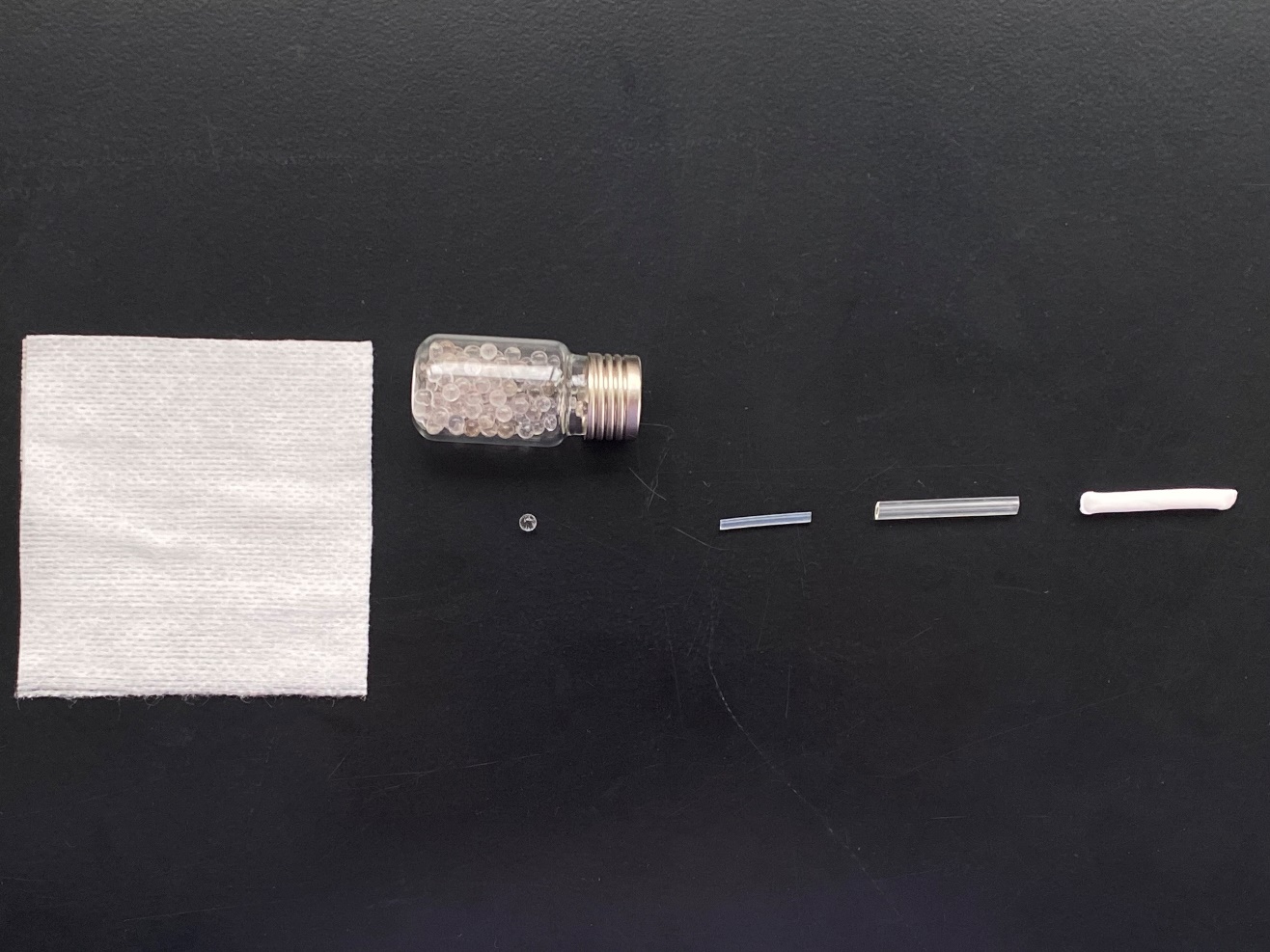


**Fig. S2** TD-GC×GC/ToFMS contour plot chromatograms of the emissions of the sampling system trapped on PSP (left) and PowerSorb® (right)


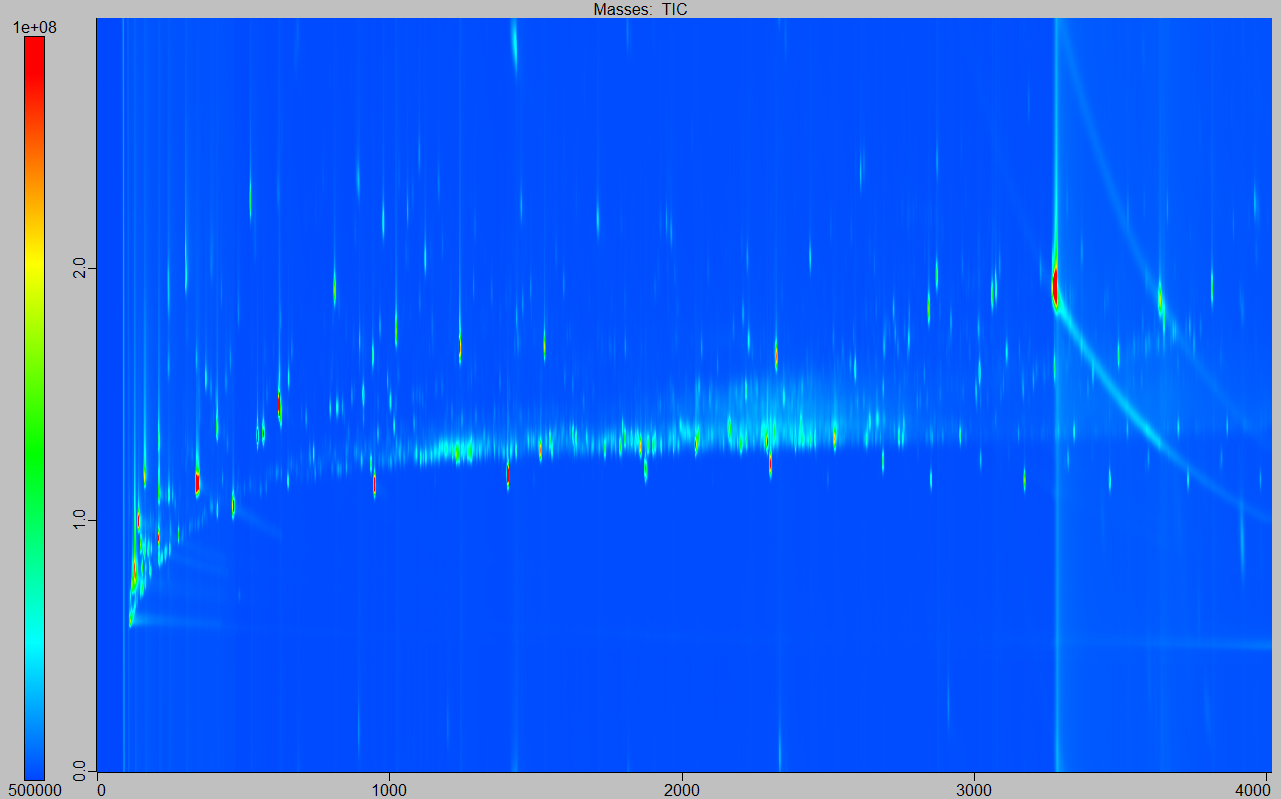

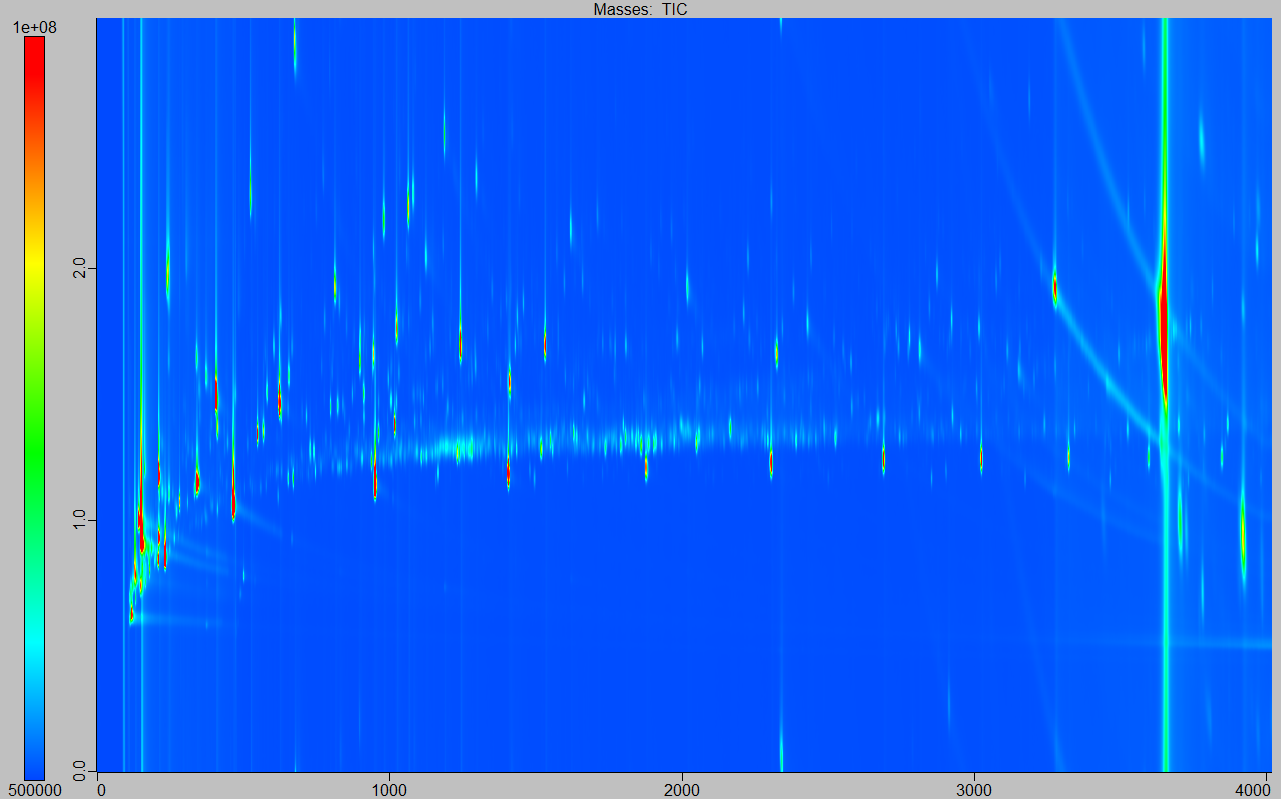


**Table S2.** List of peaks detected and tentatively identified in real body odor samples taken with both PowerSorb® and PSP.

| Peak Name | RT 1D | RT 2D | Area | Selected mass for integration |
| --- | --- | --- | --- | --- |
| Carbon disulfide | 140.991 | 0.715 | 60140819 | XIC(75.95±500ppm) |
| Butanal, 3-methyl- | 203.987 | 0.986 | 134140218 | XIC(44.04±500ppm) |
| 1-Butanol | 209.987 | 1.111 | 1155062940 | XIC(56.08±500ppm) |
| Butanal, 2-methyl- | 212.986 | 0.998 | 151418413 | XIC(57.08±500ppm) |
| Sulfide, allyl methyl | 245.984 | 1.002 | 54159688 | XIC(88.03±500ppm) |
| 1,3,6-Trioxocane | 257.983 | 1.099 | 242394358 | XIC(88.05±500ppm) |
| Acetoin | 257.983 | 1.404 | 1249340156 | XIC(45.06±500ppm) |
| 1-Propene, 1-(methylthio)-, (Z)- | 296.981 | 1.094 | 69872232 | XIC(44.99±500ppm) |
| Peak 159 | 452.971 | 1.291 | 9347293 | XIC(114.07±500ppm) |
| Butanoic acid, 3-methyl- | 515.967 | 2.165 | 167376509 | XIC(60.03±500ppm) |
| Butanoic acid, 2-methyl- | 542.965 | 2.123 | 122551905 | XIC(74.04±500ppm) |
| Peak 185 | 650.958 | 1.439 | 11910169 | XIC(72.07±500ppm) |
| 2(5H)-Furanone | 683.956 | 2.933 | 285311420 | XIC(55.03±500ppm) |
| 1,3,5,7-Tetroxane | 689.956 | 1.993 | 18970105 | XIC(61.05±500ppm) |
| 2-Nonene, 3-methyl-, (E)- | 788.95 | 1.23 | 242605652 | XIC(55.07±500ppm) |
| 2-Octene, 3,7-dimethyl-, (Z)- | 809.948 | 1.24 | 72427050 | XIC(70.08±500ppm) |
| 3-Nonene, 3-methyl-, (E)- | 815.948 | 1.226 | 198428963 | XIC(55.07±500ppm) |
| 2-Octene, 3,7-dimethyl-, (Z)- | 845.946 | 1.238 | 159558592 | XIC(70.09±500ppm) |
| β-Myrcene | 911.942 | 1.338 | 28871893 | XIC(93.07±500ppm) |
| 2-Propanol, 1,1'-oxybis- | 998.936 | 2.54 | 1144838291 | XIC(45.05±500ppm) |
| Benzyl alcohol | 1031.93 | 2.354 | 122222666 | XIC(79.06±500ppm) |
| 1-Propanol, 3,3'-oxybis- | 1052.93 | 2.679 | 986843650 | XIC(59.06±500ppm) |
| Propanoic acid, 2-methoxy-, methyl ester | 1067.93 | 2.643 | 833335569 | XIC(59.06±500ppm) |
| 1,3,6-Octatriene, 3,7-dimethyl-, (Z)- | 1079.93 | 1.391 | 4601035 | XIC(93.07±500ppm) |
| 1-Propanol, 3,3'-oxybis- | 1121.93 | 2.37 | 198949355 | XIC(59.07±500ppm) |
| 1-Octanol | 1145.93 | 1.771 | 115431957 | XIC(55.07±500ppm) |
| 1-Propanol, 3,3'-oxybis- | 1154.93 | 2.774 | 157897500 | XIC(59.06±500ppm) |
| Cyclohexene, 1-methyl-4-(1-methylethylidene)- | 1190.92 | 1.404 | 4251953 | XIC(93.07±500ppm) |
| Benzene, 1-methyl-4-(1-methylethenyl)- | 1193.92 | 1.573 | 12745898 | XIC(117.07±500ppm) |
| 3-Octanol, 3,7-dimethyl- | 1223.92 | 1.641 | 389382347 | XIC(55.07±500ppm) |
| Peak 297 | 1226.92 | 1.711 | 20907732 | XIC(93.07±500ppm) |
| α-Terpineol | 1484.9 | 1.822 | 15318707 | XIC(59.07±500ppm) |
| Peak 340 | 1547.9 | 1.965 | 43721178 | XIC(69.08±500ppm) |
| 2-Octene, 2,6-dimethyl- | 1604.9 | 1.501 | 1255703497 | XIC(55.09±500ppm) |
| 2-Propanol, 1-(2-butoxy-1-methylethoxy)- | 1646.89 | 1.722 | 12865854 | XIC(59.07±500ppm) |
| Bicyclo[3.1.1]heptane, 6,6-dimethyl-3-methylene- | 1685.89 | 1.608 | 80824908 | XIC(93.07±500ppm) |
| Cyclohexene, 1-methyl-5-(1-methylethenyl)- | 1916.88 | 1.645 | 123090242 | XIC(93.07±500ppm) |
| 1,5,5-Trimethyl-6-methylene-cyclohexene | 1922.88 | 1.634 | 30780058 | XIC(93.07±500ppm) |
| 4-Hexen-1-ol, 5-methyl-2-(1-methylethenyl)-, acetate | 2003.87 | 1.618 | 132016851 | XIC(69.08±500ppm) |
| Peak 406 | 2063.87 | 1.268 | 14833876 | XIC(73.06±500ppm) |
| 1H-3a,7-Methanoazulene, 2,3,4,7,8,8a-hexahydro-3,6,8,8-tetramethyl-, [3R-(3α,3aβ,7β,8aα)]- | 2075.87 | 1.511 | 16564076 | XIC(119.08±500ppm) |
| Peak 412 | 2117.86 | 1.927 | 24032506 | XIC(105.07±500ppm) |
| cis-Thujopsene | 2123.86 | 1.529 | 23509851 | XIC(119.09±500ppm) |
| 5,9-Undecadien-2-one, 6,10-dimethyl-, (Z)- | 2174.86 | 1.764 | 52543781 | XIC(69.08±500ppm) |
| Dodecane, 1-chloro- | 2219.86 | 1.521 | 997508042 | XIC(57.09±500ppm) |
| 1-Dodecanol | 2225.86 | 1.724 | 713818839 | XIC(55.07±500ppm) |
| 4H-Inden-4-one, 1,2,3,5,6,7-hexahydro-1,1,2,3,3-pentamethyl- | 2285.85 | 1.959 | 423404905 | XIC(191.14±500ppm) |
| Peak 443 | 2300.85 | 1.584 | 69551695 | XIC(115.05±500ppm) |
| Naphthalene, 1,2,3,4-tetrahydro-1,1,2,4,4,7-hexamethyl- | 2315.85 | 1.578 | 13713068 | XIC(201.17±500ppm) |
| Peak 451 | 2336.85 | 1.719 | 233375677 | XIC(57.08±500ppm) |
| Peak 452 | 2354.85 | 1.721 | 229080301 | XIC(57.08±500ppm) |
| Peak 461 | 2405.85 | 1.609 | 27100593 | XIC(115.06±500ppm) |
| Peak 464 | 2420.85 | 1.74 | 91223677 | XIC(57.08±500ppm) |
| Peak 466 | 2426.84 | 1.539 | 16648309 | XIC(88.05±500ppm) |
| Peak 472 | 2441.84 | 1.742 | 70371586 | XIC(57.08±500ppm) |
| Peak 474 | 2456.84 | 1.272 | 4612268 | XIC(73.06±500ppm) |
| Dodecanoic acid, ethyl ester | 2510.84 | 1.547 | 45408964 | XIC(88.05±500ppm) |
| Cedrol | 2525.84 | 1.859 | 81019003 | XIC(119.08±500ppm) |
| Peak 488 | 2534.84 | 1.535 | 16968586 | XIC(101.06±500ppm) |
| Peak 492 | 2567.84 | 1.639 | 36764550 | XIC(85.07±500ppm) |
| Peak 495 | 2582.83 | 1.622 | 52850986 | XIC(99.06±500ppm) |
| Peak 502 | 2618.83 | 1.538 | 16157524 | XIC(101.06±500ppm) |
| Peak 504 | 2627.83 | 1.56 | 58191230 | XIC(103.07±500ppm) |
| 1-Iodo-2-methylundecane | 2642.83 | 1.583 | 97578633 | XIC(57.09±500ppm) |
| Cyclopentaneacetic acid, 3-oxo-2-pentyl-, methyl ester | 2642.83 | 2.069 | 44427574 | XIC(83.05±500ppm) |
| Amberonne (isomer 1) | 2645.83 | 1.835 | 80071866 | XIC(119.08±500ppm) |
| Amberonne (isomer 3) | 2663.83 | 1.838 | 1301329526 | XIC(191.19±500ppm) |
| Benzene, (1-ethylnonyl)- | 2666.83 | 1.491 | 98613930 | XIC(91.05±500ppm) |
| Amberonne (isomer 3) | 2675.83 | 1.869 | 458147293 | XIC(191.18±500ppm) |
| Tetradecane, 1-chloro- | 2690.83 | 1.518 | 405647270 | XIC(57.09±500ppm) |
| 1-Tetradecanol | 2690.83 | 1.685 | 321902688 | XIC(55.07±500ppm) |
| Amberonne (isomer 3) | 2711.83 | 1.89 | 51814417 | XIC(69.08±500ppm) |
| Peak 534 | 2729.83 | 1.547 | 28286998 | XIC(88.05±500ppm) |
| Peak 535 | 2729.83 | 1.876 | 226135882 | XIC(135.11±500ppm) |
| Benzene, (1-methyldecyl)- | 2744.82 | 1.517 | 108661737 | XIC(105.07±500ppm) |
| 1-Nonadecanamine, N,N-dimethyl- | 2759.82 | 1.372 | 287721418 | XIC(58.08±500ppm) |
| Peak 552 | 2783.82 | 1.654 | 37042419 | XIC(57.05±500ppm) |
| Peak 559 | 2801.82 | 1.638 | 33464150 | XIC(59.07±500ppm) |
| Peak 560 | 2801.82 | 1.78 | 18906300 | XIC(137.13±500ppm) |
| Naphtho[2,1-b]furan, dodecahydro-3a,6,6,9a-tetramethyl- | 2822.82 | 1.782 | 7045385 | XIC(221.19±500ppm) |
| 1-Chloro-2-dodecyloxyethane | 2828.82 | 1.578 | 674376914 | XIC(57.09±500ppm) |
| Benzene, (1-propylnonyl)- | 2837.82 | 1.486 | 96552364 | XIC(91.05±500ppm) |
| Octanal, 2-(phenylmethylene)- | 2840.82 | 1.922 | 917826690 | XIC(91.05±500ppm) |
| Peak 583 | 2849.82 | 1.735 | 21476014 | XIC(213.17±500ppm) |
| Naphtho[2,1-b]furan, dodecahydro-3a,6,6,9a-tetramethyl- | 2858.82 | 1.817 | 152802042 | XIC(221.19±500ppm) |
| Peak 589 | 2861.82 | 1.686 | 51232444 | XIC(185.14±500ppm) |
| Tetradecanoic acid, ethyl ester | 2864.82 | 1.54 | 76697817 | XIC(88.05±500ppm) |
| Ethanone, 1-[(3R,3aR,7R,8aS)-2,3,4,7,8,8a-hexahydro-3,6,8,8-tetramethyl-1H-3a,7-methanoazulen-5-yl]- | 2891.81 | 1.927 | 312135693 | XIC(161.09±500ppm) |
| (R)-(-)-14-Methyl-8-hexadecyn-1-ol | 2909.81 | 1.85 | 232672713 | XIC(81.07±500ppm) |
| Tetradecanoic acid, ethyl ester | 2939.81 | 1.545 | 235552313 | XIC(88.05±500ppm) |
| Benzene, (1-methylundecyl)- | 2960.81 | 1.517 | 106495569 | XIC(105.07±500ppm) |
| Peak 628 | 2978.81 | 1.711 | 857630761 | XIC(229.16±500ppm) |
| Peak 631 | 2984.81 | 1.717 | 1011449413 | XIC(229.16±500ppm) |
| Peak 641 | 3017.81 | 1.628 | 793621287 | XIC(99.07±500ppm) |
| Peak 647 | 3026.81 | 1.751 | 52577549 | XIC(229.16±500ppm) |
| Peak 651 | 3041.81 | 1.751 | 21479001 | XIC(229.16±500ppm) |
| Benzene, (1-propyldecyl)- | 3044.81 | 1.487 | 52466113 | XIC(91.05±500ppm) |
| 7-Acetyl-6-ethyl-1,1,4,4-tetramethyltetralin | 3059.8 | 1.766 | 4595505548 | XIC(243.27±500ppm) |
| Peak 658 | 3074.8 | 1.73 | 57023770 | XIC(214.17±500ppm) |
| Benzene, (1-ethylundecyl)- | 3089.8 | 1.503 | 59125770 | XIC(91.06±500ppm) |
| 7-Acetyl-6-ethyl-1,1,4,4-tetramethyltetralin | 3104.8 | 1.793 | 237130272 | XIC(243.17±500ppm) |
| Peak 669 | 3116.8 | 1.79 | 183750767 | XIC(243.17±500ppm) |
| Ethanamine, 2-hydrazino-N,N-dimethyl-2-oxo- | 3134.8 | 1.615 | 523933258 | XIC(58.08±500ppm) |
| Pentadecanoic acid, ethyl ester | 3140.8 | 1.543 | 61018048 | XIC(88.05±500ppm) |
| Peak 673 | 3143.8 | 1.788 | 167378101 | XIC(243.17±500ppm) |
| Peak 681 | 3167.8 | 1.793 | 136816837 | XIC(243.17±500ppm) |
| Ethanol, 2-(tetradecyloxy)- | 3188.8 | 1.706 | 85145299 | XIC(57.09±500ppm) |
| Oxacycloheptadec-8-en-2-one, (8Z)- | 3200.8 | 1.855 | 77542517 | XIC(67.07±500ppm) |
| Octane, 1,1'-oxybis- | 3275.79 | 1.608 | 606250555 | XIC(57.09±500ppm) |
| Ethyl 9-hexadecenoate | 3281.79 | 1.589 | 35329603 | XIC(55.07±500ppm) |
| Hexadecanoic acid, ethyl ester | 3332.79 | 1.548 | 111142044 | XIC(88.05±500ppm) |
| Ethylene brassylate | 3362.78 | 2.073 | 75544588 | XIC(55.07±500ppm) |
| Peak 715 | 3368.78 | 1.657 | 724434170 | XIC(57.09±500ppm) |
| N-Dimethylaminomethyl-tert.-butyl-isopropylphosphine | 3509.78 | 1.61 | 201982694 | XIC(58.08±500ppm) |
| Heneicosane | 3521.77 | 1.361 | 22477231 | XIC(57.09±500ppm) |
| 1-Nonadecanamine, N,N-dimethyl- | 3620.77 | 1.669 | 179816716 | XIC(58.08±500ppm) |
| Diethylene glycol monododecyl ether | 3692.76 | 1.764 | 89832266 | XIC(57.09±500ppm) |
| Peak 766 | 3845.75 | 1.734 | 219635023 | XIC(57.09±500ppm) |
| 1-Propanamine, 3-dibenzo[b,e]thiepin-11(6H)-ylidene-N,N-dimethyl-, S-oxide | 3953.75 | 1.669 | 33965718 | XIC(58.08±500ppm) |

**Table S3.** List of peaks detected and tentatively identified only in real body odor sample taken with PSP.

| Peak name | 1D RT | 2D RT | Area | Selected mass for integration |
| --- | --- | --- | --- | --- |
| Peak 8 | 110.993 | 2.563 | 2742405 | XIC(118.01±500ppm) |
| Peak 16 | 128.992 | 0.658 | 32557744 | XIC(55.07±500ppm) |
| Oxalic acid | 134.991 | 1.151 | 1199905554 | XIC(46.02±500ppm) |
| Propanenitrile, 3-hydroxy- | 137.991 | 0.82 | 91967630 | XIC(53.04±500ppm) |
| Peak 36 | 143.991 | 0.879 | 13056534 | XIC(57.05±500ppm) |
| 2-Butanone | 146.991 | 0.802 | 23003128 | XIC(72.06±500ppm) |
| 1-Propanol | 146.991 | 0.868 | 15458528 | XIC(59.06±500ppm) |
| Butanal | 161.99 | 0.865 | 59369235 | XIC(44.04±500ppm) |
| Peak 59 | 164.989 | 0.809 | 78299842 | XIC(155.01±500ppm) |
| 1,3-Dioxolane, 2-methyl- | 200.987 | 0.936 | 28979720 | XIC(73.04±500ppm) |
| 2-Pentanone | 233.985 | 1.097 | 296725021 | XIC(86.07±500ppm) |
| Propanoic acid | 257.983 | 1.62 | 1294971846 | XIC(45.01±500ppm) |
| 1H-Pyrrole, 1-methyl- | 305.98 | 1.253 | 7228795 | XIC(81.06±500ppm) |
| Propanoic acid, 2-methyl- | 338.978 | 1.828 | 33692749 | XIC(73.04±500ppm) |
| 1,3-Dioxolane, 2-ethyl-4-methyl- | 380.976 | 1.189 | 25664166 | XIC(87.04±500ppm) |
| 1,3-Dioxolane, 2-ethyl-4-methyl- | 398.974 | 1.217 | 14545376 | XIC(87.04±500ppm) |
| Butanoic acid | 398.974 | 1.999 | 477706501 | XIC(60.03±500ppm) |
| Crotonic acid | 404.974 | 2.064 | 204161225 | XIC(86.03±500ppm) |
| Pyrazine, methyl- | 458.971 | 1.484 | 13518110 | XIC(94.05±500ppm) |
| 1H-Pyrrole, 2-methyl- | 503.968 | 2.104 | 5514799 | XIC(80.05±500ppm) |
| Phenylpropiolic acid | 587.962 | 1.502 | 3048816 | XIC(102.04±500ppm) |
| Oxime-, methoxy-phenyl-_ | 674.957 | 2.869 | 30683334 | XIC(133.01±500ppm) |
| Pyrazine, 2,3-dimethyl- | 695.955 | 1.623 | 6588500 | XIC(67.06±500ppm) |
| Formamide, N,N-diethyl- | 746.952 | 2.222 | 16092104 | XIC(58.08±500ppm) |
| Peak 201 | 755.952 | 1.989 | 3292647 | XIC(59.07±500ppm) |
| Peak 202 | 776.95 | 1.787 | 3823936 | XIC(211.01±500ppm) |
| Hexanoic acid | 896.943 | 2.348 | 305266062 | XIC(60.04±500ppm) |
| Benzene, (1-methylpropyl)- | 959.939 | 1.473 | 6208079 | XIC(105.07±500ppm) |
| Benzyl chloride | 968.938 | 1.772 | 66407797 | XIC(91.05±500ppm) |
| D-Limonene | 1016.93 | 1.371 | 239800571 | XIC(68.07±500ppm) |
| Eucalyptol | 1022.93 | 1.458 | 4182660 | XIC(81.07±500ppm) |
| Peak 255 | 1061.93 | 2.307 | 8354510 | XIC(60.04±500ppm) |
| Peak 261 | 1094.93 | 1.461 | 5407103 | XIC(131.05±500ppm) |
| 2,5-Hexanediol, 2,5-dimethyl- | 1103.93 | 2.439 | 55236178 | XIC(59.07±500ppm) |
| Peak 264 | 1106.93 | 1.495 | 4487169 | XIC(131.05±500ppm) |
| Octane, 1-chloro- | 1112.93 | 1.48 | 8070385 | XIC(91.03±500ppm) |
| Acetophenone | 1121.93 | 2.039 | 218237229 | XIC(105.03±500ppm) |
| Peak 273 | 1133.93 | 1.51 | 3499866 | XIC(117.04±500ppm) |
| Ethanone, 2,2-dihydroxy-1-phenyl- | 1139.93 | 2.075 | 6774676 | XIC(77.05±500ppm) |
| Peak 275 | 1145.93 | 1.706 | 33448186 | XIC(59.07±500ppm) |
| Peak 277 | 1148.93 | 1.257 | 10762247 | XIC(73.06±500ppm) |
| Peak 290 | 1199.92 | 0.172 | 119596736 | XIC(44.04±500ppm) |
| Dipropylene glycol (isomer 3) | 1202.92 | 2.568 | 5159331 | XIC(59.07±500ppm) |
| 1,3,8-p-Menthatriene | 1259.92 | 1.5 | 7204467 | XIC(91.06±500ppm) |
| Peak 307 | 1277.92 | 2.278 | 81220700 | XIC(137.04±500ppm) |
| Ethanone, 2,2-dihydroxy-1-phenyl- | 1418.91 | 2.088 | 5577278 | XIC(105.03±500ppm) |
| Benzoic acid | 1436.91 | 2.865 | 784929759 | XIC(105.03±500ppm) |
| Peak 328 | 1439.91 | 1.375 | 7949771 | XIC(71.06±500ppm) |
| Octanoic acid | 1454.91 | 2.234 | 125022020 | XIC(60.04±500ppm) |
| 2-Coumaranone | 1595.9 | 2.484 | 5308812 | XIC(78.06±500ppm) |
| 2-Propanol, 1-(2-butoxy-1-methylethoxy)- | 1631.9 | 1.721 | 9878630 | XIC(59.07±500ppm) |
| (-)-Carvone | 1631.9 | 1.958 | 9077209 | XIC(82.05±500ppm) |
| Peak 356 | 1667.89 | 1.462 | 11226305 | XIC(59.07±500ppm) |
| Linalyl acetate | 1670.89 | 1.576 | 49971278 | XIC(93.07±500ppm) |
| Cyclohexanemethanol, 4-(1-methylethyl)-, trans- | 1679.89 | 1.893 | 9564686 | XIC(95.09±500ppm) |
| Decane, 1-chloro- | 1694.89 | 1.51 | 11176656 | XIC(55.07±500ppm) |
| Cyclohexanemethanol, 4-(1-methylethyl)-, cis- | 1706.89 | 1.913 | 38357112 | XIC(95.08±500ppm) |
| 1-Decanol | 1712.89 | 1.748 | 17178120 | XIC(55.07±500ppm) |
| Bicyclo[3.1.1]heptane, 6,6-dimethyl-3-methylene- | 1751.89 | 1.702 | 6993354 | XIC(93.07±500ppm) |
| Indole | 1766.89 | 2.929 | 8308432 | XIC(117.06±500ppm) |
| ortho tert-Butyl cyclohexyl acetate | 1769.89 | 1.673 | 13703916 | XIC(57.09±500ppm) |
| Peak 371 | 1787.89 | 1.782 | 44838104 | XIC(55.07±500ppm) |
| Formamide, N,N-dibutyl- | 1790.89 | 2.18 | 39674644 | XIC(72.06±500ppm) |
| Undecanal | 1805.88 | 1.691 | 9852968 | XIC(55.07±500ppm) |
| 1,2-Benzenedicarboxylic acid | 1811.88 | 2.947 | 131601058 | XIC(104.03±500ppm) |
| Peak 375 | 1820.88 | 1.804 | 21347784 | XIC(55.07±500ppm) |
| 1,2-Benzenedicarboxylic acid | 1823.88 | 2.911 | 22535106 | XIC(104.03±500ppm) |
| Peak 378 | 1829.88 | 1.79 | 16896876 | XIC(55.07±500ppm) |
| Peak 384 | 1877.88 | 1.477 | 2512146 | XIC(47.03±500ppm) |
| Benzoic acid, 2-(hydroxymethyl)- | 1901.88 | 2.944 | 33032340 | XIC(105.03±500ppm) |
| Ethanol, 2-(3,3-dimethylcyclohexylidene)-, (Z)- | 1934.88 | 1.681 | 14353037 | XIC(79.06±500ppm) |
| Bicyclo[3.1.1]heptane, 6,6-dimethyl-2-methylene-, (1S)- | 1955.87 | 1.615 | 6084680 | XIC(93.07±500ppm) |
| Ethanol, 2-(3,3-dimethylcyclohexylidene)-, (Z)- | 1964.87 | 1.685 | 7768300 | XIC(79.06±500ppm) |
| n-Decanoic acid | 1964.87 | 2.137 | 49199549 | XIC(60.04±500ppm) |
| Diphenyl ether | 2042.87 | 1.798 | 3480234 | XIC(170.07±500ppm) |
| Dodecanal | 2066.87 | 1.685 | 30473601 | XIC(57.07±500ppm) |
| Peak 410 | 2099.87 | 1.632 | 57037060 | XIC(99.06±500ppm) |
| 1H-Isoindole-1,3(2H)-dione, 2-(hydroxymethyl)- | 2192.86 | 0.07 | 2432288 | XIC(76.04±500ppm) |
| Undecanoic acid | 2204.86 | 2.091 | 15113624 | XIC(60.04±500ppm) |
| α Isomethyl ionone | 2240.86 | 1.832 | 9807856 | XIC(135.08±500ppm) |
| 3-Ethyl-4,4-dimethyl-2-(2-methylpropenyl)cyclohex-2-enone | 2243.86 | 1.926 | 7992235 | XIC(177.13±500ppm) |
| Peak 434 | 2249.86 | 1.925 | 2025897 | XIC(63.96±500ppm) |
| Vinyl lauryl ether | 2291.85 | 1.438 | 81174313 | XIC(57.08±500ppm) |
| Peak 446 | 2303.85 | 1.613 | 6664437 | XIC(132.09±500ppm) |
| Dibenzofuran | 2312.85 | 1.983 | 7571442 | XIC(168.06±500ppm) |
| Formic acid, dodecyl ester | 2363.85 | 1.594 | 38720818 | XIC(55.07±500ppm) |
| Benzene, (1-butylhexyl)- | 2378.85 | 1.473 | 36960883 | XIC(91.05±500ppm) |
| Peak 458 | 2393.85 | 1.537 | 6891588 | XIC(101.06±500ppm) |
| Benzene, (1-propylheptyl)- | 2396.85 | 1.48 | 28405615 | XIC(91.05±500ppm) |
| Dodecane, 1-bromo- | 2420.85 | 1.555 | 34136896 | XIC(57.09±500ppm) |
| Peak 467 | 2429.84 | 1.724 | 9434113 | XIC(66.06±500ppm) |
| Benzene, (1-ethyloctyl)- | 2438.84 | 1.486 | 25073066 | XIC(91.06±500ppm) |
| Dodecanoic acid | 2438.84 | 2.038 | 84039429 | XIC(60.04±500ppm) |
| Peak 478 | 2507.84 | 1.605 | 9848521 | XIC(69.05±500ppm) |
| Diethyl Phthalate | 2507.84 | 2.167 | 24391839 | XIC(149.03±500ppm) |
| Cycloheptane, 4-methylene-1-methyl-2-(2-methyl-1-propen-1-yl)-1-vinyl- | 2513.84 | 1.873 | 18939630 | XIC(81.08±500ppm) |
| Peak 482 | 2516.84 | 1.693 | 12490374 | XIC(58.06±500ppm) |
| Pentanoic acid, 2,2,4-trimethyl-3-carboxyisopropyl, isobutyl ester | 2516.84 | 1.726 | 67712310 | XIC(71.06±500ppm) |
| Benzene, (1-methylnonyl)- | 2519.84 | 1.515 | 32256446 | XIC(105.07±500ppm) |
| Hexadecane | 2522.84 | 1.322 | 212065560 | XIC(57.09±500ppm) |
| Cycloheptane, 4-methylene-1-methyl-2-(2-methyl-1-propen-1-yl)-1-vinyl- | 2540.84 | 1.862 | 28644564 | XIC(81.08±500ppm) |
| Peak 491 | 2555.84 | 1.83 | 9052085 | XIC(191.18±500ppm) |
| Benzophenone | 2579.83 | 2.148 | 19766871 | XIC(105.03±500ppm) |
| Benzene, (1-pentylhexyl)- | 2591.83 | 1.476 | 42820512 | XIC(91.05±500ppm) |
| Peak 497 | 2591.83 | 1.599 | 155646161 | XIC(59.06±500ppm) |
| Oxalic acid, allyl hexadecyl ester | 2597.83 | 1.689 | 168934703 | XIC(57.08±500ppm) |
| Benzene, (1-butylheptyl)- | 2600.83 | 1.471 | 111023939 | XIC(91.05±500ppm) |
| Amberonne (isomer 1) | 2600.83 | 1.834 | 19571732 | XIC(119.09±500ppm) |
| Benzene, (1-propyloctyl)- | 2621.83 | 1.482 | 79013504 | XIC(91.05±500ppm) |
| Tridecanoic acid | 2657.83 | 2.009 | 7162220 | XIC(60.04±500ppm) |
| 1,3-Dioxolane, 2-pentadecyl- | 2675.83 | 1.562 | 54353659 | XIC(73.03±500ppm) |
| n-Hexyl salicylate | 2690.83 | 1.803 | 11719325 | XIC(120.02±500ppm) |
| 1,1'-Biphenyl, 2,2',5,5'-tetramethyl- | 2693.83 | 1.747 | 14233933 | XIC(195.12±500ppm) |
| Peak 528 | 2708.83 | 1.74 | 3513680 | XIC(195.12±500ppm) |
| Benzene, 1,1'-(1,2-cyclobutanediyl)bis-, trans- | 2723.83 | 1.838 | 73512746 | XIC(104.06±500ppm) |
| 2-Pentadecanone | 2735.82 | 1.693 | 81193912 | XIC(58.06±500ppm) |
| 1-Tetradecanamine, N,N-dimethyl- | 2750.82 | 1.387 | 517122134 | XIC(58.08±500ppm) |
| 4-(1,1-Dimethylheptyl)phenol | 2753.82 | 2.227 | 22269209 | XIC(135.08±500ppm) |
| Peak 543 | 2756.82 | 1.893 | 10258016 | XIC(203.18±500ppm) |
| Peak 545 | 2759.82 | 1.5 | 2183109 | XIC(229.20±500ppm) |
| Peak 546 | 2759.82 | 1.751 | 6819655 | XIC(195.12±500ppm) |
| Peak 548 | 2771.82 | 1.563 | 6150718 | XIC(59.07±500ppm) |
| 4-(7-Methyloctyl)phenol | 2771.82 | 2.223 | 15928169 | XIC(135.08±500ppm) |
| Peak 550 | 2774.82 | 1.719 | 355545927 | XIC(57.08±500ppm) |
| Peak 551 | 2780.82 | 2.22 | 4359191 | XIC(121.07±500ppm) |
| 4-(7-Methyloctyl)phenol | 2789.82 | 2.206 | 11762955 | XIC(135.08±500ppm) |
| 2,6-Diisopropylnaphthalene | 2792.82 | 1.743 | 3762682 | XIC(197.13±500ppm) |
| Methyl tetradecanoate | 2795.82 | 1.554 | 7390010 | XIC(74.05±500ppm) |
| Ethanone, 2-bromo-1,2-diphenyl- | 2798.82 | 2.255 | 17232701 | XIC(105.03±500ppm) |
| Benzene, (1-pentylheptyl)- | 2804.82 | 1.472 | 123798474 | XIC(91.05±500ppm) |
| Benzene, (1-butyloctyl)- | 2813.82 | 1.476 | 98351820 | XIC(91.05±500ppm) |
| Peak 564 | 2813.82 | 1.906 | 8154620 | XIC(120.02±500ppm) |
| Peak 566 | 2819.82 | 1.508 | 2459638 | XIC(215.18±500ppm) |
| Peak 572 | 2831.82 | 2.209 | 5808552 | XIC(149.10±500ppm) |
| Peak 580 | 2843.82 | 1.841 | 675762259 | XIC(104.06±500ppm) |
| Hexestrol, O-(n-propyloxycarbonyl)- | 2852.82 | 2.212 | 32873265 | XIC(135.08±500ppm) |
| Peak 592 | 2867.82 | 2.225 | 6640564 | XIC(149.10±500ppm) |
| 1,3-Dioxolane, 2-heptyl- | 2870.82 | 1.506 | 9080483 | XIC(73.04±500ppm) |
| Tetradecanoic acid | 2870.82 | 1.978 | 109208334 | XIC(73.04±500ppm) |
| Dodecanamide | 2870.82 | 2.434 | 223921683 | XIC(59.05±500ppm) |
| Tetradecane, 1-bromo- | 2873.82 | 1.562 | 8473696 | XIC(57.09±500ppm) |
| Benzene, (1-ethyldecyl)- | 2882.82 | 1.498 | 84908618 | XIC(91.05±500ppm) |
| Peak 599 | 2882.82 | 1.589 | 6568981 | XIC(59.07±500ppm) |
| Anthracene | 2888.82 | 2.16 | 7450393 | XIC(178.08±500ppm) |
| Ethyl 9-tetradecenoate | 2891.81 | 1.586 | 8012063 | XIC(55.07±500ppm) |
| Octadecane, 1-isocyanato- | 2912.81 | 1.625 | 5492650 | XIC(99.07±500ppm) |
| Peak 616 | 2918.81 | 1.99 | 8507616 | XIC(129.07±500ppm) |
| Octadecane | 2951.81 | 1.342 | 182902945 | XIC(57.09±500ppm) |
| Peak 621 | 2951.81 | 1.683 | 9464918 | XIC(58.06±500ppm) |
| 2-Ethylhexyl salicylate | 2963.81 | 1.77 | 13808523 | XIC(120.02±500ppm) |
| Ethanedione, diphenyl- | 2969.81 | 2.287 | 11217342 | XIC(105.03±500ppm) |
| Hexadecane, 2,6,10,14-tetramethyl- | 2981.81 | 1.34 | 6224489 | XIC(57.09±500ppm) |
| Isopropyl myristate | 3005.81 | 1.519 | 25805359 | XIC(60.04±500ppm) |
| Peak 636 | 3005.81 | 1.611 | 12987395 | XIC(57.09±500ppm) |
| Benzene, (1-pentyloctyl)- | 3008.81 | 1.476 | 105974539 | XIC(91.05±500ppm) |
| Peak 638 | 3011.81 | 1.237 | 7641722 | XIC(73.06±500ppm) |
| Peak 642 | 3017.81 | 1.749 | 22689311 | XIC(229.16±500ppm) |
| Benzene, (1-butylnonyl)- | 3020.81 | 1.484 | 79416823 | XIC(91.05±500ppm) |
| Oxalic acid, allyl octadecyl ester | 3023.81 | 1.685 | 50305273 | XIC(57.09±500ppm) |
| Peak 648 | 3032.81 | 1.545 | 12235414 | XIC(101.06±500ppm) |
| Eicosane, 10-methyl- | 3035.81 | 1.341 | 13606520 | XIC(57.09±500ppm) |
| Muscone | 3038.81 | 1.882 | 15055464 | XIC(55.07±500ppm) |
| Pentadecanoic acid | 3071.8 | 1.941 | 15943152 | XIC(60.04±500ppm) |
| Peak 661 | 3083.8 | 1.551 | 8032380 | XIC(88.05±500ppm) |
| 1,2-Benzenedicarboxylic acid, bis(2-methylpropyl) ester | 3086.8 | 2.012 | 70994361 | XIC(149.02±500ppm) |
| 1,3-Dioxolane-2-methanol | 3098.8 | 1.562 | 34935505 | XIC(73.04±500ppm) |
| Hexadecane, 1-chloro- | 3110.8 | 1.531 | 33654023 | XIC(57.09±500ppm) |
| 1-Hexadecanol | 3110.8 | 1.668 | 33282922 | XIC(55.07±500ppm) |
| Benzene, (1-methyldodecyl)- | 3167.8 | 1.519 | 196511357 | XIC(105.07±500ppm) |
| Octanamide, N-allyl- | 3191.8 | 2.146 | 50970508 | XIC(57.08±500ppm) |
| Hexadecanoic acid, methyl ester | 3200.8 | 1.56 | 129756121 | XIC(74.04±500ppm) |
| Peak 692 | 3206.79 | 1.64 | 8406647 | XIC(57.09±500ppm) |
| Peak 693 | 3212.79 | 1.584 | 4885525 | XIC(55.07±500ppm) |
| Ethanol, 2-(tetradecyloxy)- | 3236.79 | 1.575 | 254401679 | XIC(57.09±500ppm) |
| Peak 698 | 3260.79 | 1.543 | 5548643 | XIC(88.05±500ppm) |
| Dibutyl phthalate | 3266.79 | 2.038 | 148866661 | XIC(149.02±500ppm) |
| n-Hexadecanoic acid | 3281.79 | 1.925 | 3155513096 | XIC(73.03±500ppm) |
| Dodecanamide | 3293.79 | 2.288 | 20723642 | XIC(59.05±500ppm) |
| Benzene, (1-methyltridecyl)- | 3362.78 | 1.522 | 18855602 | XIC(105.07±500ppm) |
| 1-Octadecanol | 3491.78 | 1.659 | 32085651 | XIC(55.07±500ppm) |
| 2,3-Diphenylmaleic anhydride | 3518.77 | 2.647 | 5408156 | XIC(178.09±500ppm) |
| Peak 729 | 3545.77 | 1.69 | 6123589 | XIC(67.07±500ppm) |
| Methyl stearate | 3569.77 | 1.562 | 87380943 | XIC(74.04±500ppm) |
| Octadecanoic acid | 3635.77 | 1.877 | 411813629 | XIC(73.04±500ppm) |
| Dodecanamide | 3659.77 | 2.239 | 49697250 | XIC(59.05±500ppm) |
| Peak 753 | 3725.76 | 1.657 | 166931872 | XIC(57.09±500ppm) |
| [1,1':3',1''-Terphenyl]-2'-ol | 3737.76 | 2.137 | 6772581 | XIC(246.11±500ppm) |
| N-Methyl-N-benzyltetradecanamine | 3896.75 | 1.562 | 17564330 | XIC(134.10±500ppm) |
